# Ectopic expression of specific GA2 oxidase mutants promotes yield and stress tolerance in rice

**DOI:** 10.1111/pbi.12681

**Published:** 2017-03-23

**Authors:** Shuen‐Fang Lo, Tuan‐Hua David Ho, Yi‐Lun Liu, Mirng‐Jier Jiang, Kun‐Ting Hsieh, Ku‐Ting Chen, Lin‐Chih Yu, Miin‐Huey Lee, Chi‐yu Chen, Tzu‐Pi Huang, Mikiko Kojima, Hitoshi Sakakibara, Liang‐Jwu Chen, Su‐May Yu

**Affiliations:** ^1^ Institute of Molecular Biology Academia Sinica Nankang Taipei Taiwan, ROC; ^2^ Agricultural Biotechnology Center National Chung Hsing University Taichung Taiwan, ROC; ^3^ Institute of Plant and Microbial Biology Academia Sinica Taipei Taiwan, ROC; ^4^ Department of Life Sciences National Chung Hsing University Taichung Taiwan, ROC; ^5^ Institute of Molecular Biology National Chung Hsing University Taichung Taiwan, ROC; ^6^ Department of Plant Pathology National Chung Hsing University Taichung Taiwan, ROC; ^7^ RIKEN Center for Sustainable Resource Science Yokohama Kanagawa Japan

**Keywords:** rice, gibberellin, GA 2 oxidase 6, plant architecture, yield, stress tolerance, photosynthesis rate

## Abstract

A major challenge of modern agricultural biotechnology is the optimization of plant architecture for enhanced productivity, stress tolerance and water use efficiency (WUE). To optimize plant height and tillering that directly link to grain yield in cereals and are known to be tightly regulated by gibberellins (GAs), we attenuated the endogenous levels of GAs in rice via its degradation. GA 2‐oxidase (GA2ox) is a key enzyme that inactivates endogenous GAs and their precursors. We identified three conserved domains in a unique class of C_20_ GA2ox, GA2ox6, which is known to regulate the architecture and function of rice plants. We mutated nine specific amino acids in these conserved domains and observed a gradient of effects on plant height. Ectopic expression of some of these GA2ox6 mutants moderately lowered GA levels and reprogrammed transcriptional networks, leading to reduced plant height, more productive tillers, expanded root system, higher WUE and photosynthesis rate, and elevated abiotic and biotic stress tolerance in transgenic rice. Combinations of these beneficial traits conferred not only drought and disease tolerance but also increased grain yield by 10–30% in field trials. Our studies hold the promise of manipulating GA levels to substantially improve plant architecture, stress tolerance and grain yield in rice and possibly in other major crops.

## Introduction

Rice is a major staple crop feeding more of the human population than any other crop, and increase in rice yield is crucial for meeting the world's demand for food production in the next several decades. However, rice production has plateaued in major rice growing countries (IRRI, 2010), and global climate changes, such as rising temperature and water scarcity, further aggravate the stability of rice production. The development of new strategies for breeding rice that maintains high productivity in an adverse environment remains a major challenge.

The grain yield potential in rice is determined by both genetic and environmental factors. Plant architectures such as height, tiller number and root system are important target traits for rice breeding. The rice tiller is a specialized grain‐bearing branch that normally arises from the axil of each leaf and grows independently of the mother stem (culm) with its own adventitious roots. GAs control germination, plant height, tillering, root growth, flowering and seed production (Hussien *et al*., 2014; Lo *et al*., 2008; Yamaguchi, 2008). Thus, maintenance of optimal levels of bioactive GAs is important for plant growth and development. Slight reductions in GA levels result in semi‐dwarfed plants that are more lodging‐resistant in association with an improvement of harvest index (HI) (Khush, 1999). Manipulation of two such genes, *Reduced height 1* (*Rht1*), encoding a wheat GA signalling factor, and *semi‐dwarf* (*sd1*), encoding a rice GA biosynthesis enzyme, combined with N‐fertilizer application, led to a quantum leap of yield in semi‐dwarf cultivars of the respective plants. This provided the basis for the so‐called Green Revolution between the 1960s and 1990s (Botwright *et al*., 2005; Peng *et al*., 1999).

Genetic, biochemical and structural studies have significantly advanced our knowledge on biochemical pathways of GA biosynthesis and catabolism, genes and enzymes involved in these pathways, and the molecular mechanism of GA signalling in plants (Hedden and Thomas, 2012; Yamaguchi, 2008). Bioactive GA (GA_1_, GA_3_, GA_4_ and GA_7_) concentrations are maintained by the balanced activities of GA 3‐oxidases (GA3oxs) and GA 20‐oxidases (GA20oxs), essential enzymes regulating GA biosynthesis, and GA 2‐oxidases (GA2oxs), necessary for GA inactivation (Sun, 2008; Yamaguchi, 2008). The levels of bioactive GAs can be reduced by the commonly found class C_19_ GA2oxs, which hydroxylate the C‐2 of active C_19_‐GAs (GA_1_ and GA_4_) or C_19_‐GA precursors (GA_9_ and GA_20_), and the class C_20_ GA2oxs, which specifically hydroxylate C_20_‐GA precursors (GA_12_ and GA_53_) (Lo *et al*., 2008; Sakamoto *et al*., 2003; Yamaguchi, 2008). Class C_20_ GA2oxs contain three unique and conserved amino acid motifs that are absent in class C_19_ GA2oxs (Lee and Zeevaart, 2005; Lo *et al*., 2008), and the function of these domains is not well understood. Ectopic expression of a C_20_ GA2ox and GA2ox6 mutants deleting the conserved domain III generates semi‐dwarf plants with increased tillers and root system in transgenic rice (Lo *et al*., 2008).

Ectopic expression of GA biosynthesis and catabolism enzymes has been used to control the endogenous bioactive GA level in transgenic plants. Reduction in GA level could be accomplished by attenuating either the GA biosynthesis enzymes or those involved in its degradation. It has recently been reported that ectopic expression of a cytochrome P450 monooxygenase gene from *Populus trichocarpa* leads to the suppression of GA biosynthesis genes causing the reduction in shoot growth and improvement in tolerance to salt stress in transgenic rice (Wang *et al*., 2016). However, knocking down of GA biosynthesis enzymes does not always lead to substantial reduction in GA level due to the presence of multiple gene families for these biosynthesis enzymes (Coles *et al*., 1999; Rieu *et al*., 2008). On the other hand, simple over‐expression of a GA degradation enzyme can achieve the goal of effectively reducing GA level (Lo *et al*., 2008). However, constitutive overexpression of GA2ox enzymes often leads to severe dwarfism and sterility in transgenic plants (Sakai *et al*., 2003; Sakamoto *et al*., 2004). By overexpressing GA2ox1 under the control of a GA‐inducible GA biosynthesis gene (*GA3ox2*) promoter, semi‐dwarf transgenic rice with normal flowering and grain development can be obtained (Sakamoto *et al*., 2003).

Tillering is a crucial agronomic trait directly linked to the number of panicles, which in turn is one of the most important determinants of grain yield (Yang and Hwa, 2008). Plant height is also an important agronomic trait that is tightly associated with the HI and yield potential (Yang and Hwa, 2008). Manipulation of GA levels holds a promise for further improvement of plant architecture and increase in grain yield, as limitation of plant height usually coincides with the increase in the number of dwarfed tillers that bear panicles and grains (Lo *et al*., 2008). However, excessive tillering diverts nutrients, and tillers emerge late in the growing season may have high frequency of incompletely filled grains. Additionally, plant height is negatively correlated with tiller number, which in turn exhibits a trade‐off with filled grains. Consequently, to maximize the grain yield, optimum tiller number and plant height are important targets of rice breeding. However, there is no available information concerning the manipulation of plant height and tiller number to maximize the grain yield in cereals by modulating the level of endogenous GA.

The Green Revolution has had a profound impact in world agriculture in the last few decades, and it is credited with saving over a billion people from starvation. However, the success of Green Revolution was achieved through laborious breeding and selection for high‐yield semi‐dwarf varieties, and most currently cultivated crops have not yet reached their optimal architectures, stress tolerances and grain yields. In this study, we were able to adjust the architecture by ectopic expression of modified GA2ox6. We discovered that point mutation of certain amino acids of GA2ox6 conferred extraordinary beneficial traits such as semi‐dwarfism, increase in grain yield and enhancement of abiotic and biotic stress tolerance in transgenic rice.

## Results

### Key amino acids for functions of C_20_ GA2oxs

**Figure 1:**
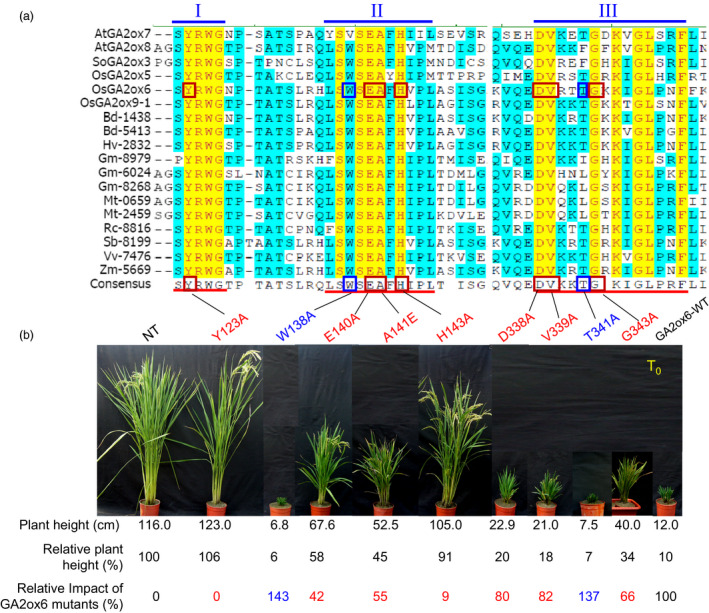
Three conserved motifs essential for function of C_20_ GA2oxs in controlling plant height. (a) Amino acid sequence alignment of C_20_ GA2oxs from different plant species. Roman numerals above the sequences indicate the three unique and conserved motifs present in C_20_ GA2oxs. Identical and conserved amino acid residues are highlighted in yellow and blue, respectively. Red underlines denote the conserved 30 amino acids in motifs I, II and III of C_20_ GA2oxs. Point mutations were introduced into the rice GA2ox6 (OsGA2ox6). Mutations that reduced or enhanced GA2ox6 impacts in transgenic rice are marked by red or blue squares, respectively. (b) Three‐month‐old T0 plants of nontransformed control (NT) and transgenic lines overexpressing various GA2ox6 mutants. The impact of GA2ox6‐WT on plant height in transgenic lines was set as 100%, and the impact of other GA2ox6 mutants was calculated relative to this value.

A total of 18 putative C_20_ GA2ox genes identified from 11 different plant species encode proteins containing three conserved motifs (Figures 1a and S1, Table S1). Sixteen amino acids in these motifs were identical, including motif I, ^**S**^/_P_YRWG; motif II, xS^**W**^ **/**_**V**_SEA^**F**^ **/**_**Y**_H^**I**^/_**V**_^**P**^ **/**_**I**_^**L**^ **/**_**M**_ **;** and motif III, DVxxxGxKxGLxxF (x represents less conserved amino acid) (Figure 1a). A total of 7 identical and 2 less conserved amino acid residues were selected for point mutations. Residues were substituted with alanine (A) except in the case of alanine 141, which was replaced with glutamate (E) (Figure 1b). Individual GA2ox6 mutants were expressed under the control of the *Ubi* promoter, and RT‐PCR analyses showed that they were expressed at similar levels in independent transgenic rice lines (Figure S2).

The relative plant height was then scored to estimate the impact of GA2ox6 mutations on the plant architecture. The impact by the wild‐type GA2ox6 (GA2ox6‐WT) was set as 100%, and GA2ox6 mutants were calculated relative to this value. Mutations of GA2ox6 at amino acid residues Y123A and H143A allowed nearly full recovery of plant heights, thus almost totally abolishing the impact on plant height; mutations at E140A, A141E and G343A partially restored plant heights with 42, 55 and 66% impacts, respectively; mutations at D338A and V339A had small effects on dwarfed plant heights, with 80–82% impacts, and mutations at two other amino acid residues, W138A and T341A, resulted in even shorter plant heights, compared to GA2ox6‐WT (Figure 1b). These results indicate that among the seven amino acids identified in the class C_20_ GA2ox, Y123 and H143 are most important, E140, A141 and G343 are second important, and D338 and V339 are less important for the function of GA2ox6 in controlling plant height. The discovery of the importance of Y123, E140, A141 and H143 in conserved motif I and II of GA2ox6 is consistent with a hypothesis that they could be involved in substrate binding (Lee and Zeevaart, 2005).

### GA2ox6 regulates plant architecture

**Figure 2:**
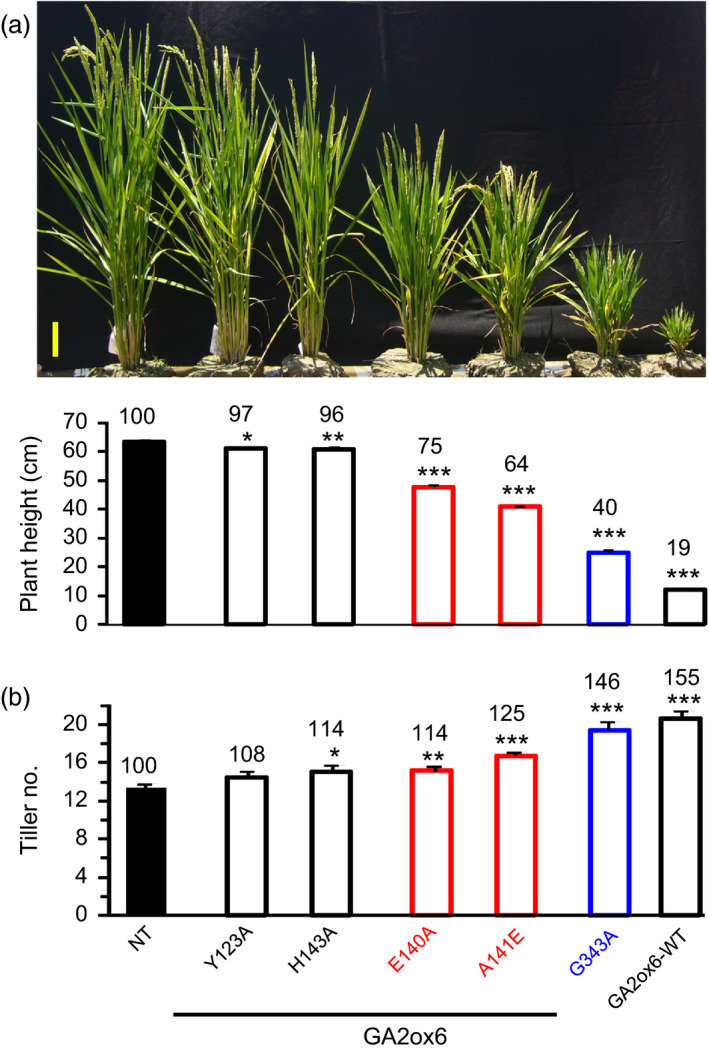
Ectopic expression of GA2ox6 and its mutants reduces plant height but increases tiller numbers in transgenic rice. Ninety‐five‐day‐old T2 transgenic plants were used in this study. (a) Morphology of NT and transgenic plants. Scale bar = 10 cm. Plant heights were quantified. (b) Tiller number. *n *= 18 for each line.

**Figure 3:**
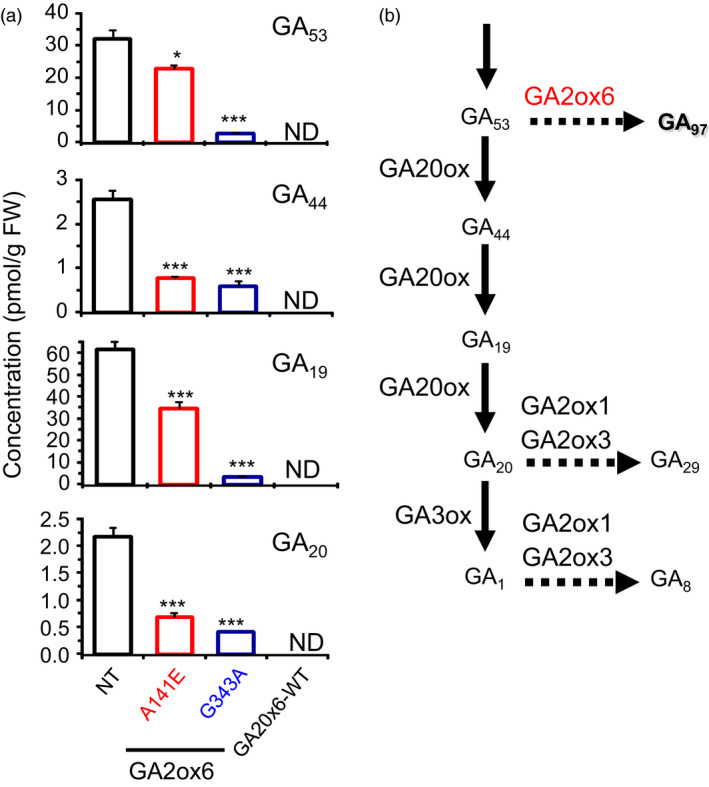
Ectopic expression of GA2ox6 and its mutants reduces the accumulation of GA precursors in transgenic lines. (a) Concentrations of GA precursors in NT and transgenic rice over‐expressing wild‐type GA2ox6 and its mutants. ND: nondetectable. (b) GA2ox6 is known to inactivate GA_53_ in the GA biosynthesis pathway. *n *= 7, 8, 8, 8 for NT and lines A141E, G343A, and GA2ox6‐WT, respectively.

Five transgenic lines expressing GA2ox6 mutants Y123A, E140A, A141E, H143A and G343A exhibited different degrees of reduction in plant heights from seedling to adult stages (Figures 2a and S3a, Table 1). In contrast, the tiller number increased inversely to plant height in lines E140A, A141E, G343A and GA2ox6‐WT (Figure 2b, Table 1). Germination rates in all transgenic lines, except GA2ox6‐WT, were similar to nontransformed control (NT) (Figure S3b). Quantitative analysis showed that levels of GA precursors such as GA_53_, GA_44_, GA_19_ and GA_20_ were significantly decreased in shoots of A141E and G343A seedlings (Figure 3). However, it is unclear why the level of GA_1_ was below the limit of detection in either NT or transgenic rice plants. To demonstrate that the different degrees of reduction in plant heights in lines A141E, G343A and GA2ox6‐WT were caused by different levels of GA deficiency; we further treated the 17‐day‐old seedlings with exogenous GA_3_ at 5 μm. The shoots of NT, A141E, G343A and GA2ox6‐WT significantly elongated after 3 days of treatment; and the transgenic plants reached to similar plant height as NT after 5 days of GA treatment (Figure S4). These observations indicate that the transgenic plants A141E, G343A and GA2ox6‐WT are likely to have different levels of endogenous GA, which can be compensated by the treatment of a high level of exogenous GA.

**Table 1:** Comparison of traits in nontransformed control (NT) and transgenic lines overexpress A141E, and G343A and GA2ox6‐WT

Traits	NT	A141E	G343A	GA2ox6‐WT
Seedling shoot length (cm) (16 DAI)	13.1 ± 0.2^a^ (100)^b^	8.3 ± 0.2 (63)	6.2 ± 0.1 (47)	4.9 ± 0.2 (37)
Seedling tiller number (16 DAI)	1.0 ± 0.0 (100)	1.24 ± 0.4 (124)	1.38 ± 0.1 (138)	1.48 ± 0.2 (148)
Seedling root number (16 DAI)	13.4 ± 0.7 (100)	15.7 ± 0.4 (117)	17.4 ± 0.4 (130)	17.4 ± 0.8 (130)
Plant height (cm) (95 DAI)	63.3 ± 0.6 (100)	40.7 ± 0.3 (64)	25.2 ± 0.8 (40)	12.3 ± 0.9 (19)
Tiller number per plant (95 DAI)	13.3 ± 0.4 (100)	16.7 ± 0.4 (125)	19.4 ± 0.9 (146)	20.6 ± 0.8 (155)
Productive tiller number per plant	10.6 ± 0.3 (100)	13.5 ± 0.3 (127)	16.2 ± 0.3 (152)	12.9 ± 0.4 (122)
Panicle length (cm)	19.8 ± 0.4 (100)	18.3 ± 0.2 (93)	15.3 ± 0.3 (78)	10.8 ± 0.2 (55)
Weight per panicle (g)	2.55 ± 0.12 (100)	2.51 ± 0.08 (98)	1.46 ± 0.07 (57)	0.61 ± 0.03 (24)
Weight of 1000 grains (g)	26.1 ± 0.2 (100)	25.8 ± 0.4 (99)	24.1 ± 0.3 (92)	23.2 ± 0.03 (89)
Fertility (%)	90.8 ± 0.01	91.6 ± 0.03	93.2 ± 0.01	93.6 ± 0.02
Total shoot weight (g)	47.1 ± 1.4 (100)	38.8 ± 0.6 (82)	25.1 ± 0.4 (53)	NA
Grain number per plant	900 ± 0.4 (100)	1130 ± 0.8 (125)	870 ± 0.7 (97)	470 ± 0.4 (53)
Grain yield (Ton/ha, 2013‐Fall)	4.22 ± 0.2 (100)	4.92 ± 0.1 (117)	3.28 ± 0.2 (78)	NA
Harvest Index (2013‐Fall) (Grain weight/shoot weight/plant)	0.55 ± 0.01 (100)	0.77 ± 0.02 (141)	0.89 ± 0.03 (161)	NA

NA, not available; DAI, days after imbibition.

a SE; *n *≧ 18 for NT, A141E, G343A, GA2ox6‐WT.

b Values in parentheses indicate % of NT.

### E140A and A141E mutants of GA2ox6 enhance grain yield

**Figure 4:**
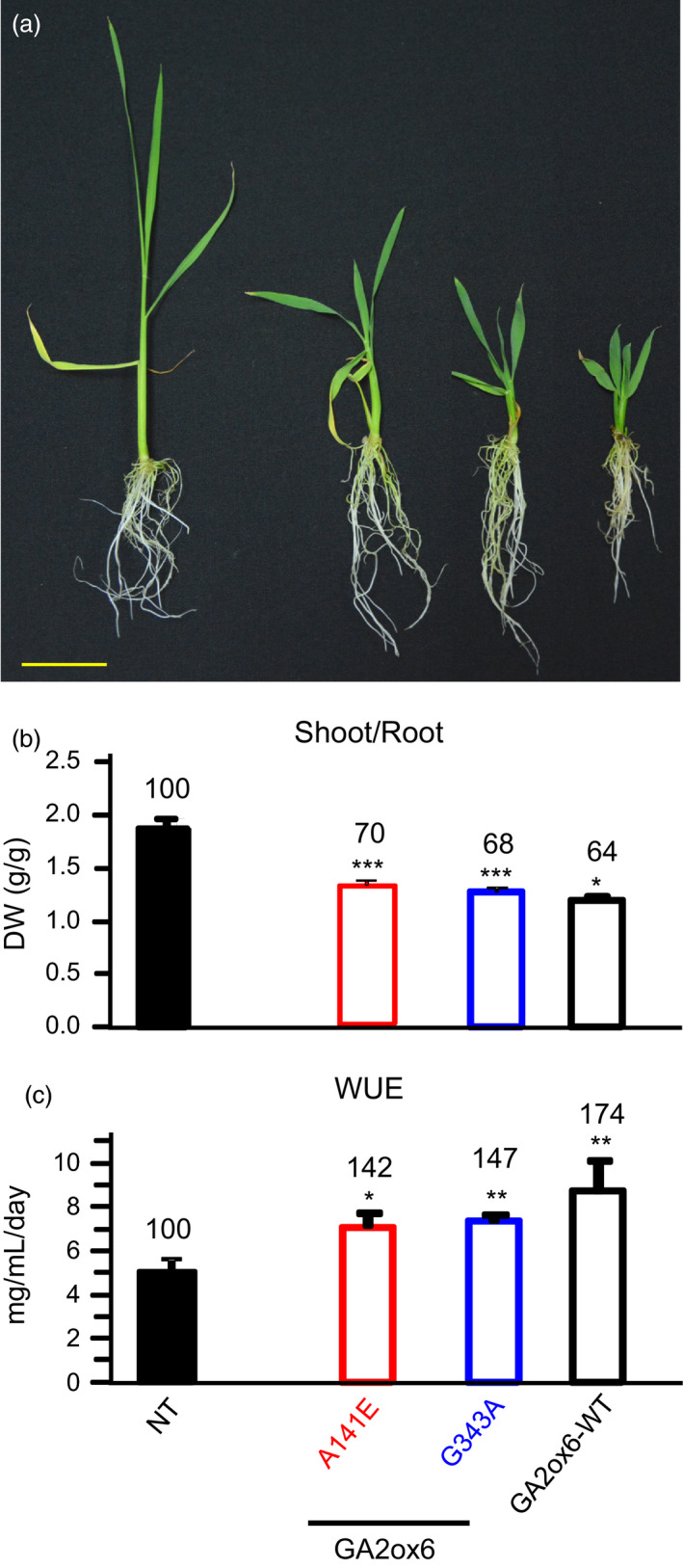
Root growth and WUE are significantly enhanced in GA‐deficient transgenic rice. (a) Morphology of 25‐day‐old plants, scale bar = 5 cm. (b) Shoot/root ratio. (c) WUE. *n *= 21 for each line.

**Figure 5:**
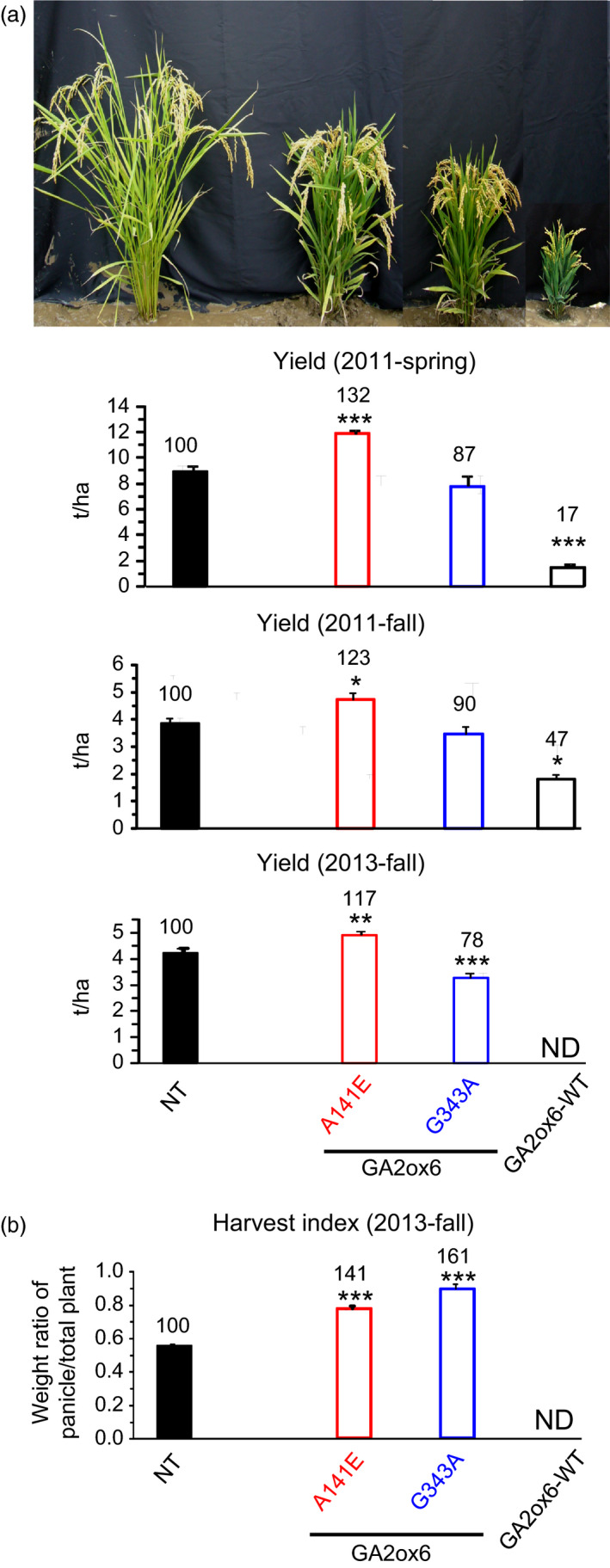
Grain yield is significantly enhanced in GA‐deficient transgenic rice. (a) Morphology of rice plants near harvest and grain yield in ton per hectare in spring and fall, 2011, and in fall, 2013. (b) Harvest index in fall, 2013; 2011‐Spring: *n *= 10 for each line; 2011‐Fall: *n *= 18, 24, 20, 27 for NT and lines A141E, G343A and GA2ox6‐WT, respectively; 2013‐Fall: *n *= 32, 55, 55, 30 for NT and lines A141E, G343A and GA2ox6‐WT, respectively. ND: not‐determined.

Three field trials in Spring and Fall of 2011, and Fall of 2013 indicated that the grain yield of line A141E was higher than other lines and NT. We also found line G343A displayed increased tolerance to various abiotic stresses compared to NT and the other transgenic lines. Consequently, transgenic lines A141E, G343A and GA2ox6‐WT were compared with NT for essential traits associated with grain yield and stress tolerance. The ratio of shoot to root dry weights decreased in both young and mature transgenic plants (Figures 4a,b and S3c). WUE also increased in transgenic young plants (Figure 4c). Importantly, the average grain yield of line A141E increased significantly by 17–32%, in contrast to those of lines G343A, which were reduced to 78–90%, as compared with NT in three separate field trials conducted during different seasons (Figures 5a and S5, Table 1). Transgenic line E140A, which was analysed later, was also found to exhibit increased average grain yield by 19% in one field trial (Figure S5). The HIs of both lines A141E and G343A were significantly increased over that of NT (Figure 5b, Table 1).

**Figure 6:**
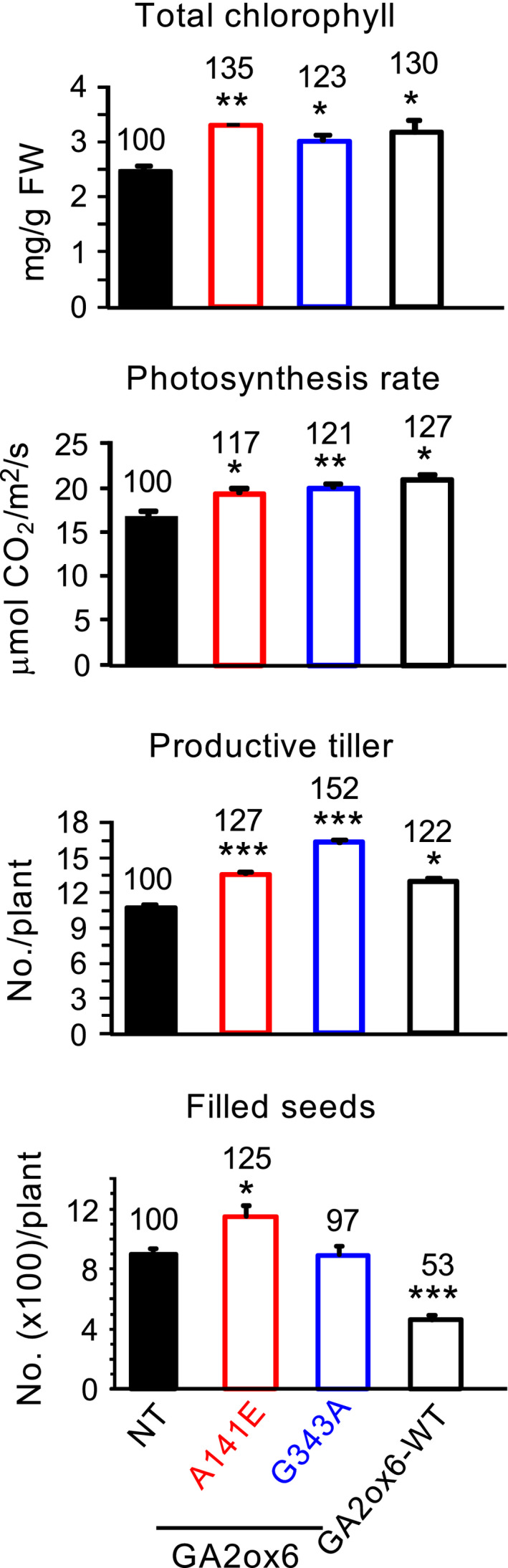
Moderate GA deficiencies improve multiple agronomic traits. Measured parameters: total chlorophyll content in 25‐day‐old plants, maximal photosynthesis rate, number of productive tillers and numbers of fertile (filled) seeds per plant before harvest. *n *= 21 for each line.

Examinations of other traits revealed that total chlorophyll, photosynthesis rate and the number of productive tillers bearing seeds were higher in transgenic lines; however, the number of filled seeds was significantly increased only in line A141E and reduced in line GA2ox6‐WT (Figure 6, Table 1). The grain and panicle weights and panicle length of line A141E were similar to NT but significantly reduced in lines G343A and GA2ox6‐WT (Figure S6, Table 1), indicating that more carbon assimilates were produced and partitioned into grains on increased tillers in line A141E. Grain morphology showed no difference in all lines (Figure S6). Line E140A displayed similar traits to line A141E mentioned above.

### A141E and G343A mutants of GA2ox6 enhance abiotic stress tolerance

**Figure 7:**
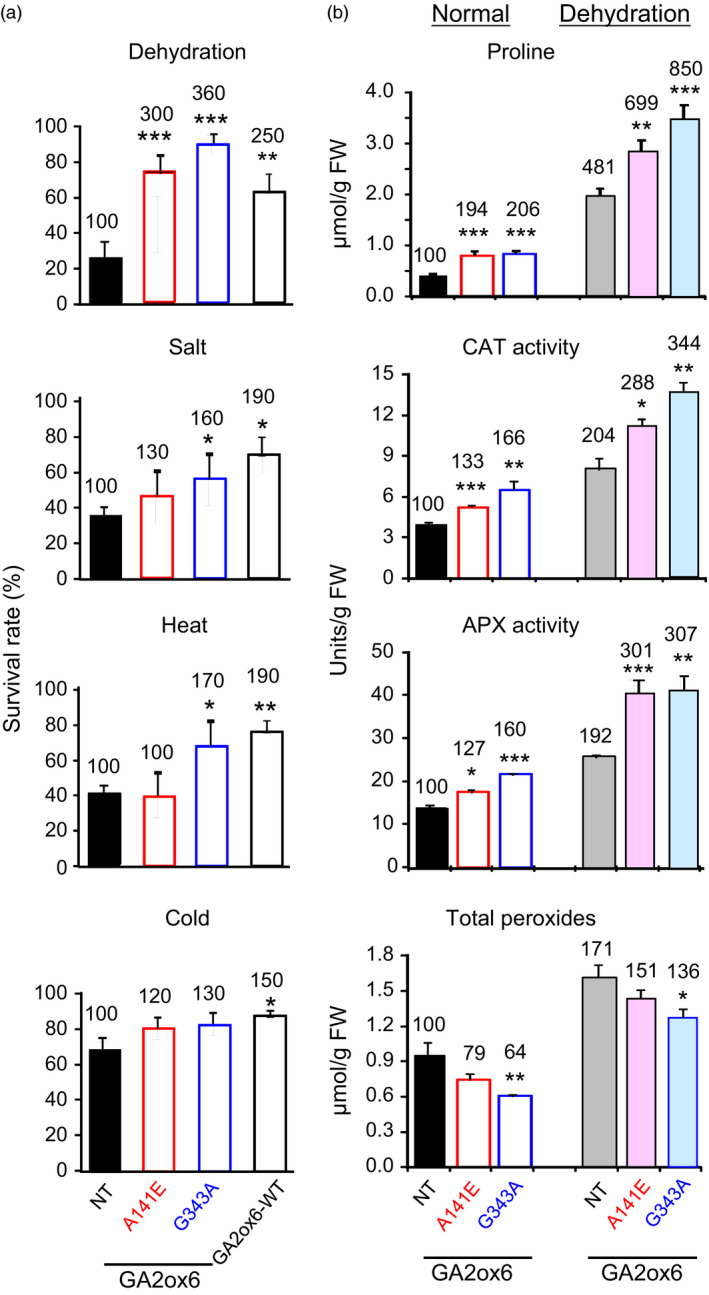
Abiotic stress tolerance, proline contents and ROS scavenging enzymes are enhanced in GA‐deficient transgenic rice plants. Fourteen‐day‐old plants were used in following experiments. (a) Survival rates after recovery from various stress treatments, *n *= 120, 49, 64 and 73 for drought treatment; *n *= 124, 74, 58 and 85 for salt treatment; *n *= 117, 44, 58 and 85 for cold treatment; and *n *= 134, 74, 69 and 56 for heat treatment, for NT and lines A141E, G343A and GA2ox6‐WT, respectively. (b) Proline content, catalase (CAT) and ascorbate peroxidase (APX) activities and total peroxide contents in plants treated with or without dehydration. *n *= 6 for each line.

A141E and G343A transgenic rice lines were tested for tolerance to various abiotic stresses. Both lines were significantly more tolerant to dehydration, and line G343A also had greater tolerance to salt and heat than NT (Figure 7a).

Accumulation of the amino acid proline is induced in many plant species in response to environmental stresses and that has been proposed to play an important role in the adjustment to osmotic and oxidative stresses caused by salt and drought (Sperdouli and Moustakas, 2012; Szabados and Savoure, 2010). As shown in Figure 7b, proline levels and CAT and APX activities were all greater in lines A141E and G343A than in NT under normal growth conditions. These factors were also increased in NT but were much greater in transgenic lines under dehydration conditions, and were generally higher in line G343A than in line A141E regardless of dehydration. In contrast, the two transgenic lines had lower total peroxide levels than NT under both normal and dehydration conditions. These findings demonstrated enhanced physiological adaptation to abiotic stresses in rice over‐expressing GA2ox6 mutants.

**Figure 8:**
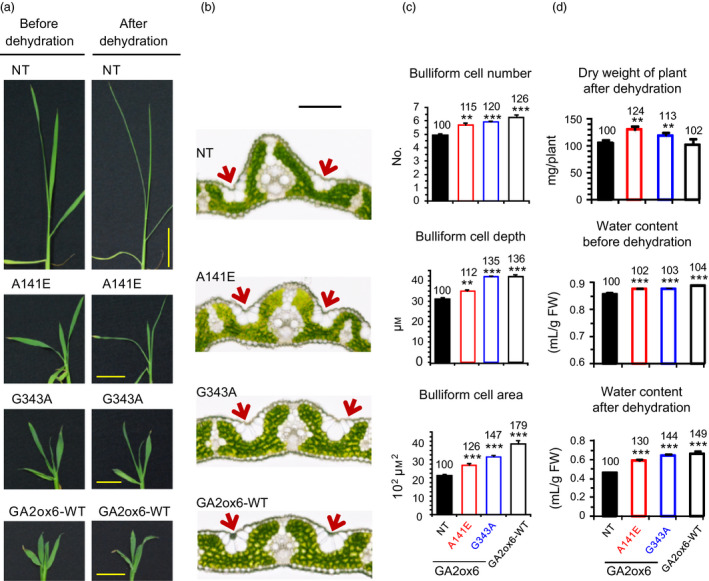
The elevated abiotic stress tolerance is associated with enhanced bulliform cell volume and water contents in GA‐deficient plants. Fourteen‐day‐old plants were used in following experiments. (a) Leaf morphology before and after 3.5‐h dehydration. Scale bar = 3 cm. Live images are also shown in Video S1. (b) Cross section of first fully expanded leaf under normal growth conditions. Scale bar = 100 μm. Arrow indicates bulliform cells. More leaf sections are also shown in Fig. S7. (c) Quantification of bulliform cell number, depth and area, *n *= 18, 14, 22, 14 for NT, A141E, G343A and GA2ox6‐WT, respectively. (d) Plant dry weight after dehydration and water contents before and after 3.5‐h dehydration. *n *= 21 for each line.

Leaf shape plays a crucial role in photosynthesis and plant development by affecting light interception, leaf temperature and water loss (O'Toole and Cruz, 1980). Leaves are shorter and wider in transgenic lines in contrast to long and narrow leaves in NT (Figure 8a). Leaves rolled and wilted significantly in NT but were less wilted in transgenic lines after dehydration (Figures 8a and S7a). After rehydration in water, leaves opened slowly in NT but rapidly in transgenic lines (Video S1). The two top young leaves of NT and lines A141E and G343A wilted during osmotic stress treatment with PEG; after recovery in water, these leaves in lines A141E and G343A expanded and continued to grow, while in NT never expanded and ultimately died (Video S2). Lines A141E and G343A grown in soil were also dehydrated. Leaves of both lines recovered completely from dehydration after re‐watering, but those of NT were severely damaged and survival rate was low (Figure S7b).

Bulliform cells (BCs) are large, bubble‐shaped epidermal cells that are present in groups on the upper surface of leaves of many grasses, and are thought to play a role in providing mechanical strength in the unfolding of developing leaves and in the rolling and unrolling of mature leaves in response to alternating dehydration and well‐watered conditions, respectively (Moore *et al*., 1998). Histological examination of leaf cross sections revealed that BCs were more prominent and extended deeper into the leaf interior (Figures 8b and S8), and the number and volume of BCs were also increased in transgenic lines as compared to NT (Figure 8c). Expanded BCs may retain more water facilitating leaf unrolling after dehydration. Indeed, transgenic lines contain more water in shoots than NT after dehydration for 3.5 h (Figure S9). In addition to shoot, the entire plants also contain more water in transgenic lines (Figure 8d).

### E140A, A141E and G343A mutants of GA2ox6 enhance disease resistance

**Figure 9:**
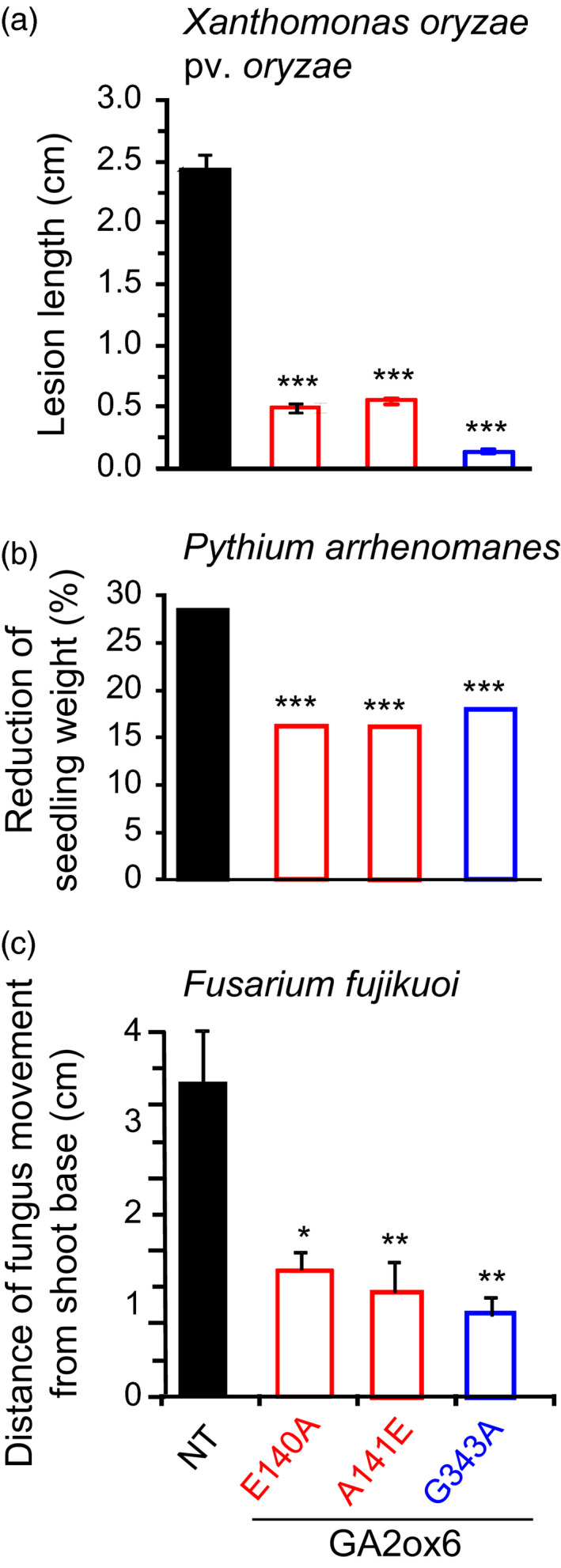
GA‐deficient transgenic rice is more resistant to pathogens. (a) Lesion expansion on leaves after infection by *Xanthomonas oryze pv. oryzae*. (b) Seedling weight after infection by *Pythium arrhenomanes*. (c) Upward migration of *F. fujikuoi* from shoot base. *n *= 10 for each line in each treatment.

Diseases are a major constraint for achieving optimal yields. Underexpression of a GA biosynthesis enzyme, GA20ox3, reduces plant height but enhances resistance to pathogens *Magnaporthe oryzae* (causing rice blast) and *Xanthomonas oryze pv. oryzae* (causing bacterial blight) in rice (Qin *et al*., 2013). We found that E140A, A141E and G343A mutants displayed smaller lesion sizes after infection with *X. oryzae pv. oryzae*, maintained greater seedling weight after infection with *Pythium arrhenomanes* (causing seedling blight), and restricted the movement of *Fusarium fujikuroi* (causing the foolish‐seedling disease) in shoots as compared with NT (Figure 9).

### Reprogramming GA‐regulated transcriptional networks

**Figure 10:**
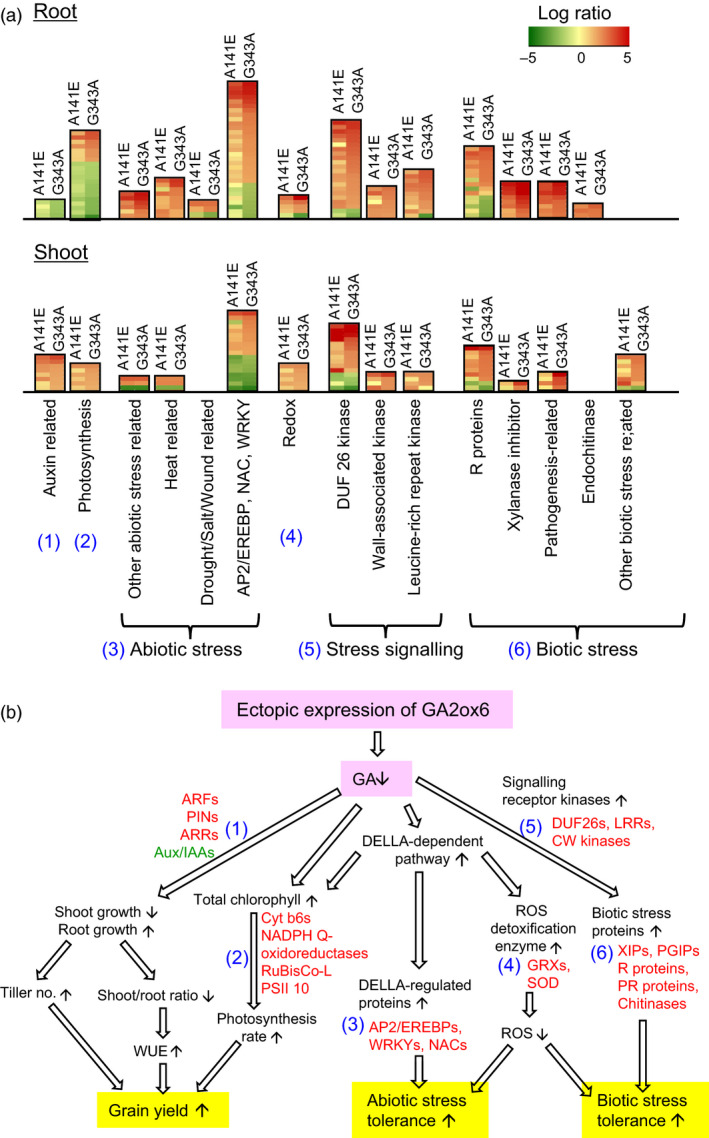
Genes that are significantly up‐regulated contribute to increase in grain yield and abiotic and biotic stress tolerance in GA‐deficient transgenic rice. (a) Expression profiles of six groups of genes (number in parenthesis labelled in blue) related to grain yield and abiotic and biotic stress tolerance were compared in roots and shoots between A141E and G343A mutants and NT. Clustered genes up‐ and down‐regulated are marked in red and green, respectively. For detailed lists of genes in each cluster and extent of changes, see Tables S2 and S3. (b) Coordinated events and pathways leading to increase in grain yield and abiotic and biotic stress tolerance. Numbers denote up‐regulated genes corresponding to gene clusters in A. Red and green fonts indicate representative up‐ and down‐regulated genes, respectively. For abbreviation of gene names, see Materials and Methods. Small upward and downward arrowheads indicate up‐ and down‐regulation of genes. Open arrowheads indicate suggested sequences of events.

GA‐deficient mutants A141E and G343A exhibited pleiotropic alterations in morphology and physiology. To better understand how the GA regulation network controlling plant growth and development, the genomewide transcriptomics profiling was performed for shoots and roots in A141E and G343A in comparison with NT. Primary responsive genes were identified and validated by microarray and RT‐PCR analyses (Figures 10 and S10–S12 and Tables S2 and S3). Based on stringent statistics and filtering (*P* values < 0.05, signal ratio changes > threefold), transcription was found to be significantly reprogrammed in two transgenic lines, with total 765 and 688 genes being up‐ and down‐regulated in roots, and 588 and 382 genes being up‐ and down‐regulated in shoots, respectively, in A141E and G343A (Figure S10a). Large sets of genes were specifically or commonly up‐ and down‐regulated in shoots and roots in A141E and G343A, with more genes being affected in G343A than in A141E and more in roots than in shoots (Figure S10b). Genes involved in abiotic and biotic stress responses and signalling were highly enriched in shoots and roots of the two lines (Figures 10a and S10c, Tables S2 and S3).

We found that up‐regulation of six groups of genes in shoots and roots of A141E and G343A may be responsible for enhanced tillering, root growth, biotic and abiotic stress tolerance and grain yield (Figure 10): (1) Group 1 genes encoding auxin response factors (ARFs), auxin efflux transporter PINs and type B Arabidopsis response regulator (ARR), which are known to promote tillering and root development in cereals (Hussien *et al*., 2014; Orman‐Ligeza *et al*., 2013), were up‐regulated, and the repressor protein Aux/IAA was down‐regulated. (2) Group 2 genes encoding RuBisCo large subunit and proteins involved in light reaction of photosynthesis, such as cytochrome b6/f, NADPH Q‐oxidoreductase and PSII 10 kDa polypeptide (PSII 10), were up‐regulated in A141E and G343A shoots, which correlates with the enhanced photosynthesis rates seen in these plants. It should be noted that the expression level of photosynthesis related genes is actually reduced in A141E and G343A in roots (Figure 10a). However, the significance of this reduction is not yet clear. (3) Group 3 genes encoding proteins involved in abiotic stress tolerance, such as AP2/EREBPs, WRKYs and NACs. (4) Group 4 genes encoding proteins responsible for ROS scavenging, such as glutaredoxins (GRXs) and superoxide dismutase (SOD), which could also be regulated by the DELLA‐dependent pathway. (5) Group 5 genes encoding numerous signalling receptor kinases, such as DUF26 kinases, cell wall (CW)‐associated kinases and leucine‐rich repeat (LRR) kinases. (6) Group 6 genes encoding proteins involved in plant defence, such as xylanase inhibitor proteins (XIPs), polygalacturonase inhibitor proteins (PGIPs), resistance (R) proteins and pathogenesis‐related (PR) proteins (Juge, 2006; Pogorelko *et al*., 2013), were highly up‐regulated.

## Discussion

To optimize plant architecture for enhancing productivity as well as stress tolerance or water saving capacity in crops is one of major challenges in modern agricultural biotechnology. In the present study, constitutive ectopic expression of GA2ox6 mutants controlling endogenous GA levels has successfully manipulated plant height, tiller number, shoot‐to‐root ratio, WUE, photosynthesis rate, stress tolerance, pathogen resistance, harvest index and grain yield in transgenic rice.

### Five amino acids are important for functions of C_20_ GA2oxs

Although the function of the class C_20_ GA2oxs in deactivation of GA precursors is known in both dicots and monocots (Lee and Zeevaart, 2005; Lo *et al*., 2008), the function of the three unique and conserved domains in this enzyme family is not well understood. Only motif II of the three domains has been proposed to function as a substrate‐binding site based on amino acid sequence similarity to the GA biosynthetic enzyme GA20ox (Lee and Zeevaart, 2005; Lo *et al*., 2008). Within these three domains, 16 of 30 amino acids are identical in GA2oxs from 11 different species, indicating their functional conservation throughout evolution (Figure 1a). However, individuals of these identical amino acids seem to play distinct as well as common functions. For example, amino acids Y123 and H143 were most important, and E140, A141 and G343 were second important, while D338 and V339 were less important, for the function of GA2ox6 in controlling plant height and tillering (Figure 1b). Interestingly, mutations in the less conserved amino acids W138 and T341 enhanced the function of GA2ox6 by causing even more dwarfed plants. Mutations of GA2ox6 at E140, A141 and G343 conferred ideal plant architecture and physiology in transgenic rice in terms of productivity and stress tolerance.

### Moderate semi‐dwarf rice mutants possess high‐yield potential

Three seasons of field trials demonstrated that transgenic line A141E significantly increased grain yield by 17–32%, and one field trial showed that line E140A also increased grain yield by 19% (Figures 5 and S5, Table 1). Yield potential is known to be controlled by complicated multiple factors. The so‐called New Plant Type (NPT) of rice, including lower tillering (9–10 tillers/plant), no unproductive tillers, 200–250 grains/panicle, dark green, thick and erect leaves, and vigorous and deep root system, has been conceptualized for further improving grain yield potential for rice post the green revolution era (Jeon *et al*., 2011). Although the architecture in lines E140A and A141E only meet certain criteria of NPT, a few newly acquired traits may contribute to the increase in its grain yield as compared with NT: first, the ratio of shoot to root dried biomass was reduced and a more vigorous root system was maintained (Figures 4a and S3c, S9), which got better access to nutrients and water in soil and thus had a potential of improving the HI (Figure 5b, Table 1). Second, dark green and erect leaves (Figure S8) with increased total chlorophyll content were developed and photosynthesis rate was enhanced. Third, more productive tillers were generated for grain production and filling (Figure 6, Table 1). No obvious negative phenotype was associated with line A141E and E140A, except slightly delayed germination rate (1‐day delay) (Figure S3b) and heading date (1‐week delay). Overexpression of an ethylene‐responsive AP2/ERF factor, EATB, suppresses the expression of GA20ox2, also leading to reduced plant height, increased tillering and higher grain yield in transgenic rice (Qi *et al*., 2011). Our studies demonstrate that lines E140A and A141E, with heights 75 and 64% of NT at adult stage, respectively (Figure 2a), possess combined traits favourable for plant growth and represent ideotypes of rice with high‐yield potentials.

### Moderate GA‐deficient mutant is more stress tolerant

GA‐deficient rice is also significantly more tolerant to dehydration and slightly more tolerant to high salinity, heat and cold than NT (Figure 7a). GA deficiency has been implicated in enhancing salt tolerance in Arabidopsis, presumably by overexpressing a stress‐responsive transcription factor encoded by the *dwarf and delayed‐flowering 1* (*ddf1*), which activates stress‐related genes such as *RD29A/COR78* (Magome *et al*., 2004), and in promoting cold and pathogen tolerance in a *gibberellin‐insensitive dwarf 1* (*gid1*) rice mutant but with unknown mechanism (Tanaka *et al*., 2006). Several factors might contribute to the capacity of abiotic stress tolerance in lines A141E and G343A. First, abiotic stresses induce the formation of toxic ROS that causes protein and membrane damages, and efficient scavenging of ROS by catalase (CAT) and ascorbate peroxidase (APX) is essential for osmotic tolerance in plants (Apel and Hirt, 2004; Hasegawa *et al*., 2000). Increase in proline levels and APX and CAT activities and decrease in ROS or peroxide accumulation may account for enhanced abiotic stress tolerance in these lines (Figure 7b). The notion is supported by a study showing that under abiotic or biotic stress, the GA‐repressible DELLA proteins are induced to activate the expression of ROS‐detoxification enzymes, thus reducing ROS levels and conferring stress tolerance in Arabidopsis (Achard *et al*., 2008). Second, higher chlorophyll levels enhance drought tolerance (Figure 6), as chlorophyll content is regarded one of several measures for capacity of dehydration tolerance in rice (Luo, 2010). Normally, chlorophyll contents declined in plants under drought conditions. Chlorophyll content, *Fv/Fm* which represents the maximum quantum yield of PSII, was reduced to less extents in drought‐tolerant than in drought‐sensitive barley varieties (Guo *et al*., 2009). Third, the higher water content in roots and leaves (Figures 8d and S9) enhance drought tolerance. Plants with higher water status could maintain physiological functions under drought conditions (Luo, 2010). Fourth, less water was consumed for the same production as NT, leading to significant increase in WUE in transgenic lines (Figure 4c). WUE is defined as the economic production per unit water consumption, but it may or may not related to drought resistance (Luo, 2010). GA‐deficient rice turns out to have both enhanced WUE and drought resistance.

It is worthwhile noting that leaf blades of transgenic lines remained relatively open, in contrast to leaves rolled adaxially quickly in NT after dehydration. The leaf water potential, and in particular, loss of turgor pressure in BCs is tightly associated with the degree of leaf rolling (O'Toole and Cruz, 1980). The expanded BC volume may account for the unrolled leaf phenotypes after dehydration, which allows photosynthesis to continue in transgenic lines under stress conditions. The number and size of BCs also affect leaf shape, as loss of function of a *Narrow Leaf 7* (*NAL7)* gene, which controls auxin biosynthesis, results in reduced size and number of BCs and leaf width in rice (Fujino *et al*., 2008). The GA‐deficient transgenic rice has shorter and wider leaf blades than NT, and such phenotype correlates with the increase in volume and number of BCs (Figure 8b,c), indicating crosstalk between GA and auxin signals may control the development of BCs and leaf width.

Although the productivity of line G343A was decreased by 10–22% in three field trials, it is more tolerant to dehydration, salinity and heat (Figure 7a). Under severe abiotic stress, enhanced stress tolerance may offset slight reduction in grain yield potential in line G343A as compared with line A141E. Transgenic line GA2ox6‐WT also exhibited similar traits to lines A141E and G343A; however, the grain yield was significantly lower; we attribute this to overproduction of tillers that compete for carbon assimilates and mineral nutrients. Further, insufficient activity of GA may also impair reproductive growth and attenuate seed production in the GA2ox‐WT line. Greater tiller number (Figure 2) and lower GA levels (Figure 3) may also explain the reduced grain yields in line G343A as compared with line A141.

### Moderate GA deficiency reprograms transcriptional networks

GA is an essential hormone regulating growth and development throughout the entire life cycle of plants. Altered expression of genes regulated by GA may impact many aspects of plant growth and developmental processes. In the present study, we mainly focused on transcriptomics relevant to grain yield, abiotic and biotic stress tolerance (Figure 10), as these traits have been foci of many research.

Balance and crosstalk of hormones control root development, for example, auxin promotes lateral root growth (Orman‐Ligeza *et al*., 2013), while GA deficiency promotes adventitious root growth (Lo *et al*., 2008). We showed that mutations at A141 and G343 resulted in down‐regulation of Aux/IAA but up‐regulation of ARFs and ARR, which may increase auxin biosynthesis and re‐direct its transportation, leading to increase in bud activity and cytokinin biosynthesis (Domagalska and Leyser, 2011; Hussien *et al*., 2014; Muller and Leyser, 2011; Orman‐Ligeza *et al*., 2013) and correlating with enhanced tiller and root growth in these two mutants.

Lower endogenous GA levels could reduce the destruction of DELLA protein (Sun, 2008), which in turn directly or indirectly induces a set of abiotic stress‐related transcription factors (Gallego‐Bartolome *et al*., 2011; Qi *et al*., 2014; Sun, 2010), such as those encoded by the group 3 genes, AP2/EREBPs, WRKYs and NACs, which are indeed up‐regulated in shoots and roots of A141E and G343A transgenic lines. We also found that some genes encoding proteins responsible for ROS scavenging, such as SOD, APX, peroxiredoxin and glutaredoxin (GRX) were up‐regulated in A141E and G343A (Tables S2 and S3), which could be related to ROS scavenging as well as tolerance to biotic and abiotic stresses (Figure 7).

GA deficiency also redirected important transcriptional networks related to biotic stress responses. One set of up‐regulated genes includes signalling molecules and receptors, such as group 5 genes encoding DUF26 kinases, CW‐associated kinases and LRR kinases. These genes together with other up‐regulated group 6 genes encoding XIPs, PGIPs, R proteins and PR proteins could be responsible for the enhanced plant defence. It is intriguing that some PGIPs and most of XIPs are strongly induced in A141E and G343A transgenic lines, as these cell wall degrading enzyme inhibitors have been shown to be induced by pathogen infection and important in plant defence as well as in cell elongation (Juge, 2006; Pogorelko *et al*., 2013). Conceivably, some PGIPs and XIPs inhibit fungal pectic enzymes and xylanases, respectively, which are needed to degrade plant cell walls for a successful invasion, and other PGIPs and XIPs inhibit endogenous pectic enzymes and xylanase activity leading to lower cell wall extensibility and dwarfism. Additionally, we observed up‐regulation of genes associated with PAMP‐triggered immunity (PTI) and effector‐triggered immunity (ETI) systems (Jones and Dangl, 2006), both of which correlate with the enhanced disease resistance in A141E and G343A.

Lines A141E and G343A behave very similarly, but also have major differences in some traits. For example, line G343A is shorter and has more tiller number and bulliform cell volume and greater abiotic stress tolerance, but with reduced grain yield as compared to line A141E. These differences in traits could be resulted from more significantly reduced levels of some GA precursors in line G343A (Figure 3), which is consistent with the observation that the expression of more genes was affected in both shoots and roots in line G343A than in line A141E (Figure S10, Tables S2 and S3) as compared to the NT. Among these genes, 7.6 and 13.9% in shoots and roots of A141E, and 10.4 and 14.1% in shoots and roots of G343A, respectively, are known to be involved in abiotic and biotic stress responses and signalling. The fold induction of ARFs and ARR, AP2/EREBPs, WRKYs and NACs involved in abiotic stress tolerance was also significantly higher in G343A than in A141A. Overexpression of stress‐related proteins may also attribute to the yield penalty in G343A as compared with A141E.

Taken together, several factors might contribute to the tolerance for water‐deficit stress in GA‐deficient transgenic lines. Elevated proline levels and ROS scavenging activities protect plants from damage under dehydration stress, and higher chlorophyll contents maintain photosynthesis under dehydration. Higher water contents may also allow plants to maintain metabolic functions under drought conditions. Finally, an increase in BC cell volume that store water may facilitate leaf unfolding and rapid recovery from dehydration. Accumulation of ROS has also been shown to correlate with the aggressiveness of pathogens, and resistant cultivars had weaker symptoms, less ROS accumulation and higher activity of the ROS scavenging system in plants (El‐Komy, 2014; Govrin and Levine, 2000). It appears that multiple defence mechanisms against pathogens are turned on in the GA‐deficient transgenic rice.

In summary, although the Green Revolution has drastically enhanced productivity of wheat and rice, our studies demonstrated that ectopic expression of GA2ox mutants can further modulate endogenous GA levels, leading to partitioning of even greater proportions of plant biomass into grains. In addition, these manipulations resulted in enhanced tolerance to both biotic and abiotic stresses. This advanced version of Green Revolution could be applied to other crops for further optimization of plant architecture and function so that more stress‐ and disease‐tolerant varieties requiring less water but with higher yields could be produced.

## Materials and methods

### Plant materials

The rice cultivar *Oryza sativa* L. cv Tainung 67 was used in this study. Transgenic and NT seeds were surface‐sterilized with 2.5% NaOCl and placed on MS agar medium (Murashige and Skoog Basal Medium, Sigma, St. Louis, MO, USA) and incubated at 28 °C with 16‐h light and 8‐h dark for 14–20 days. Plants were transplanted to a GM‐field with bird‐free screen.

### Database searching and phylogenetic analysis of C_20_ *GA2oxs*

Database searches for GA2oxs from different plant species and identification of C_20_ *GA2oxs* using the 30 amino acids present in the three unique conserved motifs in rice GA2ox6 were carried out as described (Lo *et al*., 2008). Deduced amino acid sequences of all C_19_ and C_20_ GA2oxs were aligned as described (Lo *et al*., 2008).

### Site‐directed mutation of rice GA2ox6 and rice transformation

To generate point mutations in three conserved domains of *GA2ox6*, amino acid residues Y123, W138, E140, H143, D338, V339, T341, G343 were substituted with alanine (A), and A141 was substituted with glutamate (E). Point mutations were conducted as described (Kunkel, 1985). *Ubi:GA2ox6* in plasmid pAHC18 (Lo *et al*., 2008) was used as the template for point mutations of *Ubi:GA2ox6* by QuikChange® Site‐directed Mutagenesis Kit (Stratagene, http://www.stratagene.com/) according to the manufacturer's instructions. Primers for mutagenesis are listed in Table S4. All plasmids were linearized with *Hind*III and inserted into the same site in pCAMBIA1301 (Hajdukiewicz *et al*., 1994). Resulting binary vectors were transferred into *Agrobacterium tumefaciens* strain EHA105 and subsequently used for rice transformation as described (Ho *et al*., 2000). Correct point mutations in recombinant GA2ox6 were confirmed by nucleotide sequencing of genomic DNAs isolated from transgenic lines.

### Gibberellin quantification

To measure levels of endogenous GAs, ∼200 mg of shoot tissues was collected from 21‐day‐old seedlings. Tissues were placed in a 2.0‐mL Eppendorf tube (Labcon, Petaluma, CA, USA), lyophilized and ground to powder by TissueLyser (QIAGEN, Hilden, Germany) in liquid nitrogen. The concentrations of endogenous GA compounds were analysed as described previously (Ayano *et al*., 2014; Kojima *et al*., 2009). Two biological repeats were carried out in this measurement.

### Water consumption and water use efficiency (WUE)

Eighteen‐day‐old seedlings were weighed, transferred to plastic tubes containing 50 mL water, and tube openings were sealed with parafilm. Water consumption was measured, and water was re‐filled, every 2 days for up to 8 days. The increase in fresh weight of plants during the 8‐day period, and final fresh and dried weights of plants were determined. The WUE of each plant was calculated by dividing the average increase in fresh weight by the average water consumption per day.

### Yield evaluation

Homozygosity of T3 transgenic lines was confirmed by hygromycin selection. Three‐week‐old seedlings were transplanted to the open GM‐field at National Chung‐Hsing University and grown under natural conditions. At least two repeated blocks for each transgenic and NT lines, 24 plants in each block with a layout of 3 rows X 8 lines, and the space for each plant is 25 cm × 25 cm, were designed for field tests. The plant height, matured tiller number, biomass, panicle number and total yield data were collected from 18 plants, excluding the two marginal lines, in each block.

### Chlorophyll content and photosynthetic rate

Fresh leaves were collected from the first expanded leaf of 80‐day‐old plants in the field. Leaves were ground with liquid nitrogen in a mortar and pestle. Pigments were extracted with 95% ethanol, and light absorptions at 648.6 and 664.2 nm were determined using a UV/visible spectrophotometer (Biowave II; Biochrom Ltd., Holliston, MA, USA). Concentrations of total chlorophylls were calculated as described (Lichtenthaler, 1987).

Leaf photosynthetic rate was measured from 9:30 to 16:00 using the LI‐6400 Portable Photosynthesis System with a leaf chamber fluorometer attached (Model 6400‐40; LICOR Inc., Lincoln, NE, USA); parameters used included: CO_2_ flux, 500 μm/S; block temperature, 28 °C; photosynthetically active radiation, 800 μmol photons m^2^/s. Triplicate measurements of photosynthetic assimilation rate (μM CO_2_ m^2^/S) were recorded after equilibration to a steady state (∼20 min) and with three first fully expanded leaves for each plant.

### Stress treatments

Fourteen‐day‐old plants were transferred to distilled water for one day and then incubated in a cold (4 °C) or warm (42 °C) incubator or in 200 mm NaCl solution for 2 days, or dehydrated (air‐dried) on the bench at room temperature (25–27 °C) for 6 h. All stressed plants were allowed to recover in water for 6 days in a 28 °C incubator, and survival rates were determined.

### Videos of plant responses to and recovery from stress treatments

Twenty five‐day‐old plants were treated with dehydration or 30% PEG6000 on the bench at room temperature (25–27 °C) for 3.5 h. Plants were then allowed to recover in water or roots were covered with 0.8% agar gel replenished with water at intervals for 20–24 h. Photographs were taken at an interval of 2 min. Time‐lapse videos were organized by the Windows Movie Maker v2.6.

### Quantification of proline and total peroxide content

Shoots of 14‐day‐old seedlings with or without 3‐h dehydration were weighed. Proline was extracted with a ninhydrin reagent and quantified by the absorbance at 520 nm. Proline concentrations were calculated by a calibration curve and expressed as μmol proline g‐1 fresh weight (Bates *et al*., 1973). Total peroxides of shoots of 15‐day‐old seedlings were extracted with 5% (w/v) trichloroacetic acid (TCA) according to the method described by Sagisaka (1976).

### Activity assay of antioxidant enzymes

Proteins in shoots of 14‐day‐old seedlings with or without 3‐h dehydration were extracted using the sodium phosphate buffer (50 mm, pH6.8). Activities of catalase and ascorbate peroxidase were determined by spectrophotometric methods as described by Kato and Shimizu (1985) and Nakano and Asada (1981), respectively.

### Leaf structure examination

The first fully expanded leaves of 25‐day‐old plants were collected. Cross sections of leaf blades were made with Microtome Leica VT1200 (Leica Microsystems GmbH, Wetzlor, Germany) and examined with a light microscope (Upright microscope ECLIPSE Ni‐U, Nikon Co., Tokyo, Japan).

### Water content

Seventeen‐day‐old plants were used in this experiment. For determination of water contents, shoots and roots were blotted dry with paper towels and weighed before and after dehydration (air dry) for 3.5 h. For determination of dried weight, plant materials were dried in 80 °C oven for 2 days, and dried weights of shoots and roots were measured. The water content before dehydration was calculated by subtracting dried weight from fresh weight before dehydration, and the water content after dehydration was calculated by subtracting dried weight from fresh weight after dehydration.

### Pathogen infection

Transgenic and NT seeds were surface‐sterilized with 2.5% NaOCl and dipped in running water for 3 days. Germinated seeds were infected with the following pathogens. *X. oryzae* pv. *oryzae*: germinated seeds were transferred to pots filled with peat moss, incubated at 28 °C with 16‐h light and 8‐h dark for 14 days, and transferred to pots containing field soils and peat moss (1:1) for another 21 days. Plants were then infected with bacteria at a concentration of 1 × 10^8^ cfu/mL using sterilized scissors (Kauffman *et al*., 1973). Length of lesion developed was measured. *P. arrhenomanes*: germinated seeds were transferred to 0.5X Kimura solution, pH 5.0, containing the fungus with concentration of absorbance 1.5 at OD_600_ and incubated at 28 °C with 16‐h light and 8‐h dark for 10 days. The reduction in total seedling fresh weight was measured. *F. fujikuroi*: germinated seeds were submerged in the spore suspension with concentration of 1 × 10^6^ spores/mL for 1 h. Infected seeds were transferred to 0.5X Kimura solution, pH 5.0, and incubated at 28 °C with 16‐h light and 8‐h dark for 14 days. The upward migration distance of the fungus from shoot base was measured.

### Microarray analysis

Total RNA was purified from rice shoots and roots of 17‐day‐old seedlings using Trizol® reagent (Invitrogen, Waltam, Massachusets, USA), and further purified with RNeasy Mini Kit (QIAGEN). RNA quality assessment and array experiment were conducted in the Affymetrix Gene Expression Service Lab of Academia Sinica, using the GeneChip® Rice Genome Array (Affymetrix, Taipei, Taiwan). The Affymetrix CEL files were imported into GeneSpring 12.6 software (Agilent Technol., Santa Clara, CA, USA ) for data normalization, generating the MAS5.0 algorithm. The flags of detection calls as ‘Present (P)’ or ‘Margin (M)’ were subjected to further analysis (Pepper *et al*., 2007). Genes with signal ratio greater than threefold changes were used for GeneOntology significance analysis with the AgriGO database (http://bioinfo.cau.edu.cn/agriGO), and functional classification was performed with the MapMan software (3.6.0RC1) (Thimm *et al*., 2004).

### RT‐PCR analyses

Total RNA was purified from rice leaves or roots, and RT‐PCR analyses were conducted as described (Lo *et al*., 2008)

### Primers

Nucleotides for all primers used for PCR and RT‐PCR analyses are provided in Table S4.

### Statistical analysis

All numerical data are presented as mean ± SEM (error bars indicate standard error of the mean). Statistical analyses were carried out by comparing the raw data of all individuals of each transgenic line with those of NT with the Student's *t*‐test using the SigmaPlot software (version 11.0; Systat Software, Inc., San Jose, CA, USA ).

For the relative levels shown above bars in figures, the value of each parameter in NT was set as 100, and the value of transgenic lines was calculated relative percentage to this value. Difference was compared between transgenic lines and NT. Significance levels were determined with the *t*‐test: * 0.05 > *P* ≧ 0.01, ** 0.01 > *P* ≧ 0.001, *** 0.001 > *P*.

## Authors‘ contributions

SMY, THDH and SFL involved in the designing of the project; S‐MY, T‐HDH, LJC, M‐HL, C‐YC, T‐PH and HS involved in the supervision of the project; S‐FL, K‐TH, K‐TC, M‐JJ and MK performed the experiments; M‐JJ and L‐CY provided materials and other support; S‐FL, S‐MY and T‐HDH involved in the preparation of manuscript.

## Supporting information


**Figure S1.** Phylogenetic tree analysis of GA2oxs in plants.
**Figure S2.** Ectopic expression of GA2ox6 mutants alters plant height in rice.
**Figure S3.** Plant heights are reduced, germination rates are unaltered, and shoot/root ratios are decreased in transgenic lines E140A, A141E and G343A.
**Figure S4.** Treatment with exogenous GA_3_ restores normal height in transgenic lines A141E and G343A.
**Figure S5.** Yields are increased in transgenic lines E140A and A141E in field trials.
**Figure S6.** Grain weight and morphology and panicle weight and length in transgenic line A141E are similar to those in NT.
**Figure S7.** Drought stress tolerance is enhanced in GA deficient transgenic rice.
**Figure S8.** The size of bulliform cells is expanded in GA deficient transgenic rice.
**Figure S9.** Plant volume, biomass and water content are increased in GA deficient transgenic rice.
**Figure S10.** Reprogramming of GA‐regulated transcriptional networks in GA deficient transgenic rice.
**Figure S11.** Expression of genes overrepresented in roots of GA deficient transgenic plants in response to abiotic and biotic stresses.
**Figure S12.** Expression of genes overrepresented in shoots of GA deficient transgenic plants in response to abiotic stresses.
**Table S1.** Gene names and accession numbers of GA2oxs from different plant species.
**Table S2.** GA deficiency redirected several important transcriptional networks in roots.
**Table S3.** GA deficiency redirected several important transcriptional networks in shoots.
**Table S4.** Primers used for site‐directed mutagenesis, PCR and RT‐PCR analyses and plasmid constructions.

**Video S1.** Transgenic rice over‐expressing WT and mutant GA2ox6 recovered faster from dehydration than NT.

**Video S2.** Transgenic rice over‐expressing WT and mutant GA2ox6 recovered better from osmotic stress than NT.

## References

[pbi12681-bib-0001] Achard, P. , Renou, J.P. , Berthome, R. , Harberd, N.P. and Genschik, P. (2008) Plant DELLAs restrain growth and promote survival of adversity by reducing the levels of reactive oxygen species. Curr. Biol. 18, 656–660. 18450450 10.1016/j.cub.2008.04.034

[pbi12681-bib-0002] Apel, K. and Hirt, H. (2004) Reactive oxygen species: metabolism, oxidative stress, and signal transduction. Annu. Rev. Plant Biol. 55, 373–399. 15377225 10.1146/annurev.arplant.55.031903.141701

[pbi12681-bib-0003] Ayano, M. , Kani, T. , Kojima, M. , Sakakibara, H. , Kitaoka, T. , Kuroha, T. , Angeles‐Shim, R.B. *et al*. (2014) Gibberellin biosynthesis and signal transduction is essential for internode elongation in deepwater rice. Plant, Cell Environ. 37, 2313–2324. 24891164 10.1111/pce.12377 PMC4282320

[pbi12681-bib-0004] Bates, L.S. , Waldren, R.P. and Teare, I.D. (1973) Rapid determination of free proline for water‐stress studies. Plant Soil 39, 205–207.

[pbi12681-bib-0005] Botwright, T.L. , Rebetzke, G.J. , Condon, A.G. and Richards, R.A. (2005) Influence of the gibberellin‐sensitive Rht8 dwarfing gene on leaf epidermal cell dimensions and early vigour in wheat (*Triticum aestivum* L.). Ann. Bot. 95, 631–639. 15655105 10.1093/aob/mci069 PMC4246859

[pbi12681-bib-0006] Coles, J.P. , Phillips, A.L. , Croker, S.J. , Garcia‐Lepe, R. , Lewis, M.J. and Hedden, P. (1999) Modification of gibberellin production and plant development in Arabidopsis by sense and antisense expression of gibberellin 20‐oxidase genes. Plant J. 17, 547–556. 10205907 10.1046/j.1365-313x.1999.00410.x

[pbi12681-bib-0007] Domagalska, M.A. and Leyser, O. (2011) Signal integration in the control of shoot branching. Nat. Rev. Mol. Cell Biol. 12, 211–221. 21427763 10.1038/nrm3088

[pbi12681-bib-0008] El‐Komy, M.H. (2014) Comparative analysis of defense responses in chocolate spot‐resistant and ‐susceptible faba bean (*Vicia faba*) cultivars following infection by the necrotrophic fungus *Botrytis fabae* . Plant Pathol. J. 30, 355–366. 25506300 10.5423/PPJ.OA.06.2014.0050 PMC4262288

[pbi12681-bib-0009] Fujino, K. , Matsuda, Y. , Ozawa, K. , Nishimura, T. , Koshiba, T. , Fraaije, M. and Sekiguchi, H. (2008) NARROW LEAF 7 controls leaf shape mediated by auxin in rice. Mol. Genet. Genomics 279, 499–507. 18293011 10.1007/s00438-008-0328-3

[pbi12681-bib-0010] Gallego‐Bartolome, J. , Alabadi, D. and Blazquez, M.A. (2011) DELLA‐induced early transcriptional changes during etiolated development in *Arabidopsis thaliana* . PLoS ONE 6, e23918. 21904598 10.1371/journal.pone.0023918 PMC3164146

[pbi12681-bib-0011] Govrin, E.M. and Levine, A. (2000) The hypersensitive response facilitates plant infection by the necrotrophic pathogen *Botrytis cinerea* . Curr. Biol. 10, 751–757. 10898976 10.1016/s0960-9822(00)00560-1

[pbi12681-bib-0012] Guo, P. , Baum, M. , Grando, S. , Ceccarelli, S. , Bai, G. , Li, R. , von Korff, M. *et al*. (2009) Differentially expressed genes between drought‐tolerant and drought‐sensitive barley genotypes in response to drought stress during the reproductive stage. J. Exp. Bot. 60, 3531–3544. 19561048 10.1093/jxb/erp194 PMC2724701

[pbi12681-bib-0013] Hajdukiewicz, P. , Svab, Z. and Maliga, P. (1994) The small, versatile pPZP family of Agrobacterium binary vectors for plant transformation. Plant Mol. Biol. 25, 989–994. 7919218 10.1007/BF00014672

[pbi12681-bib-0014] Hasegawa, P.M. , Bressan, R.A. , Zhu, J.K. and Bohnert, H.J. (2000) Plant cellular and molecular responses to high salinity. Annu. Rev. Plant Physiol. Plant Mol. Biol. 51, 463–499. 15012199 10.1146/annurev.arplant.51.1.463

[pbi12681-bib-0015] Hedden, P. and Thomas, S.G. (2012) Gibberellin biosynthesis and its regulation. Biochemical J. 444, 11–25. 10.1042/BJ20120245 22533671

[pbi12681-bib-0016] Ho, S.L. , Tong, W.F. and Yu, S.M. (2000) Multiple mode regulation of a cysteine proteinase gene expression in rice. Plant Physiol. 122, 57–66. 10631249 10.1104/pp.122.1.57 PMC58844

[pbi12681-bib-0017] Hussien, A. , Tavakol, E. , Horner, D.S. , Muñoz‐Amatriaín, M. , Muehlbauer, G.J. and Rossini, L. (2014) Genetics of tillering in rice and barley. Plant Genome, (accessed 13 January 2014). doi: 10.3835/plantgenome2013.10.0032.

[pbi12681-bib-0018] IRRI (2010) Rice Policy ‐ Why is it happening? http://beta.irri.org/solutions/index.php?option=com_content&task=view&id=15.

[pbi12681-bib-0019] Jeon, J.‐S. , Jung, K.‐H. , Kim, H.‐B. , Suh, J.‐P. and Khush, G. (2011) Genetic and molecular insights into the enhancement of rice yield potential. J. Plant Biol. 54, 1–9.

[pbi12681-bib-0020] Jones, J.D.G. and Dangl, J.L. (2006) The plant immune system. Nature, 444, 323–329. 17108957 10.1038/nature05286

[pbi12681-bib-0021] Juge, N. (2006) Plant protein inhibitors of cell wall degrading enzymes. Trends Plant Sci. 11, 359–367. 16774842 10.1016/j.tplants.2006.05.006

[pbi12681-bib-0022] Kato, M. and Shimizu, S. (1985) Chlorophyll metabolism in higher plants VI. Involvement of peroxidase in chlorophyll degradation. Plant Cell Physiol. 26, 1291–1301.

[pbi12681-bib-0023] Kauffman, H. , Reddy, A. , Hsieh, S. and Merca, S. (1973) Improved technique for evaluating resistance of rice varieties to *Xanthomonas oryzae* . Plant Dis. Rep. 57, 537–541.

[pbi12681-bib-0024] Khush, G.S. (1999) Green revolution: preparing for the 21st century. Genome, 42, 646–655. 10464789

[pbi12681-bib-0025] Kojima, M. , Kamada‐Nobusada, T. , Komatsu, H. , Takei, K. , Kuroha, T. , Mizutani, M. , Ashikari, M. *et al*. (2009) Highly sensitive and high‐throughput analysis of plant hormones using MS‐probe modification and liquid chromatography‐tandem mass spectrometry: an application for hormone profiling in *Oryza sativa* . Plant Cell Physiol. 50, 1201–1214. 19369275 10.1093/pcp/pcp057 PMC2709547

[pbi12681-bib-0026] Kunkel, T.A. (1985) Rapid and efficient site‐specific mutagenesis without phenotypic selection. Proc. Natl Acad. Sci. USA 82, 488–492. 3881765 10.1073/pnas.82.2.488 PMC397064

[pbi12681-bib-0027] Lee, D.J. and Zeevaart, J.A. (2005) Molecular cloning of GA 2‐oxidase3 from spinach and its ectopic expression in *Nicotiana sylvestris* . Plant Physiol. 138, 243–254. 15821147 10.1104/pp.104.056499 PMC1104179

[pbi12681-bib-0028] Lichtenthaler, H.K. (1987) [34] Chlorophylls and carotenoids: Pigments of photosynthetic biomembranes. In Methods in Enzymol ( Lester Packer, R.D. ed.), pp. 350–382. Academic Press, Elsevier, Amsterdam, Netherlands: Academic Press.

[pbi12681-bib-0029] Lo, S.F. , Yang, S.Y. , Chen, K.T. , Hsing, Y.I. , Zeevaart, J.A. , Chen, L.J. and Yu, S.M. (2008) A novel class of gibberellin 2‐oxidases control semidwarfism, tillering, and root development in rice. Plant Cell 20, 2603–2618. 18952778 10.1105/tpc.108.060913 PMC2590730

[pbi12681-bib-0030] Luo, L.J. (2010) Breeding for water‐saving and drought‐resistance rice (WDR) in China. J. Exp. Bot. 61, 3509–3517. 20603281 10.1093/jxb/erq185

[pbi12681-bib-0031] Magome, H. , Yamaguchi, S. , Hanada, A. , Kamiya, Y. and Oda, K. (2004) dwarf and delayed‐flowering 1, a novel Arabidopsis mutant deficient in gibberellin biosynthesis because of overexpression of a putative AP2 transcription factor. Plant J. 37, 720–729. 14871311 10.1111/j.1365-313x.2003.01998.x

[pbi12681-bib-0032] Moore, R. , Clark, W.D. , Vodopich, D.S. , Stern, K.R. and Lewis, R. (1998) Botany. Dubuque IA USA: McGraw‐Hill College.

[pbi12681-bib-0033] Muller, D. and Leyser, O. (2011) Auxin, cytokinin and the control of shoot branching. Ann. Bot. 107, 1203–1212. 21504914 10.1093/aob/mcr069 PMC3091808

[pbi12681-bib-0034] Nakano, Y. and Asada, K. (1981) Hydrogen peroxide is scavenged by ascorbate‐specific peroxidase in Spinach Chloroplasts. Plant Cell Physiol. 22, 867–880.

[pbi12681-bib-0035] Orman‐Ligeza, B. , Parizot, B. , Gantet, P.P. , Beeckman, T. , Bennett, M.J. and Draye, X. (2013) Post‐embryonic root organogenesis in cereals: branching out from model plants. Trends Plant Sci. 18, 459–467. 23727199 10.1016/j.tplants.2013.04.010

[pbi12681-bib-0036] O'Toole, C. and Cruz, R.T. (1980) Response of leaf water potential, stomatal resistance, and leaf rolling to water stress. Plant Physiol. 65, 428–432. 16661206 10.1104/pp.65.3.428 PMC440347

[pbi12681-bib-0037] Peng, J. , Richards, D.E. , Hartley, N.M. , Murphy, G.P. , Devos, K.M. , Flintham, J.E. , Beales, J. *et al*. (1999) ‘Green revolution’ genes encode mutant gibberellin response modulators. Nature 400, 256–261. 10421366 10.1038/22307

[pbi12681-bib-0038] Pepper, S.D. , Saunders, E.K. , Edwards, L.E. , Wilson, C.L. and Miller, C.J. (2007) The utility of MAS5 expression summary and detection call algorithms. BMC Bioinformatics, 8, 273. 17663764 10.1186/1471-2105-8-273 PMC1950098

[pbi12681-bib-0039] Pogorelko, G. , Lionetti, V. , Bellincampi, D. and Zabotina, O. (2013) Cell wall integrity: targeted post‐synthetic modifications to reveal its role in plant growth and defense against pathogens. Plant Signal. Behav. 8, e25435. 23857352 10.4161/psb.25435 PMC4002593

[pbi12681-bib-0040] Qi, W. , Sun, F. , Wang, Q. , Chen, M. , Huang, Y. , Feng, Y.Q. , Luo, X. *et al*. (2011) Rice ethylene‐response AP2/ERF factor OsEATB restricts internode elongation by down‐regulating a gibberellin biosynthetic gene. Plant Physiol. 157, 216–228. 21753115 10.1104/pp.111.179945 PMC3165871

[pbi12681-bib-0041] Qi, T. , Huang, H. , Wu, D. , Yan, J. , Qi, Y. , Song, S. and Xie, D. (2014) Arabidopsis DELLA and JAZ Proteins Bind the WD‐Repeat/bHLH/MYB complex to modulate gibberellin and jasmonate signaling synergy. Plant Cell 26, 1118–1133. 24659329 10.1105/tpc.113.121731 PMC4001373

[pbi12681-bib-0042] Qin, X. , Liu, J.H. , Zhao, W.S. , Chen, X.J. , Guo, Z.J. and Peng, Y.L. (2013) Gibberellin 20‐oxidase gene OsGA20ox3 regulates plant stature and disease development in rice. Mol. Plant‐Microbe Interact. 26, 227–239. 22992000 10.1094/MPMI-05-12-0138-R

[pbi12681-bib-0043] Rieu, I. , Ruiz‐Rivero, O. , Fernandez‐Garcia, N. , Griffiths, J. , Powers, S.J. , Gong, F. , Linhartova, T. *et al*. (2008) The gibberellin biosynthetic genes AtGA20ox1 and AtGA20ox2 act, partially redundantly, to promote growth and development throughout the Arabidopsis life cycle. Plant J. 53, 488–504. 18069939 10.1111/j.1365-313X.2007.03356.x

[pbi12681-bib-0044] Sagisaka, S. (1976) The occurrence of peroxide in a perennial plant, *Populus gelrica* . Plant Physiol. 57, 308–309. 16659472 10.1104/pp.57.2.308 PMC542013

[pbi12681-bib-0045] Sakai, M. , Sakamoto, T. , Saito, T. , Matsuoka, M. , Tanaka, H. and Kobayashi, M. (2003) Expression of novel rice gibberellin 2‐oxidase gene is under homeostatic regulation by biologically active gibberellins. J. Plant. Res. 116, 161–164. 12736788 10.1007/s10265-003-0080-z

[pbi12681-bib-0046] Sakamoto, T. , Morinaka, Y. , Ishiyama, K. , Kobayashi, M. , Itoh, H. , Kayano, T. , Iwahori, S. *et al*. (2003) Genetic manipulation of gibberellin metabolism in transgenic rice. Nat. Biotechnol. 21, 909–913. 12858182 10.1038/nbt847

[pbi12681-bib-0047] Sakamoto, T. , Miura, K. , Itoh, H. , Tatsumi, T. , Ueguchi‐Tanaka, M. , Ishiyama, K. , Kobayashi, M. *et al*. (2004) An overview of gibberellin metabolism enzyme genes and their related mutants in rice. Plant Physiol. 134, 1642–1653. 15075394 10.1104/pp.103.033696 PMC419838

[pbi12681-bib-0048] Sperdouli, I. and Moustakas, M. (2012) Interaction of proline, sugars, and anthocyanins during photosynthetic acclimation of *Arabidopsis thaliana* to drought stress. Plant Physiol. 169, 577–585. 10.1016/j.jplph.2011.12.015 22305050

[pbi12681-bib-0049] Sun, T.P. (2008) Gibberellin metabolism, perception and signaling pathways in Arabidopsis. Arabidopsis Book, 6, e0103. 22303234 10.1199/tab.0103 PMC3243332

[pbi12681-bib-0050] Sun, T.P. (2010) Gibberellin‐GID1‐DELLA: a pivotal regulatory module for plant growth and development. Plant Physiol. 154, 567–570. 20921186 10.1104/pp.110.161554 PMC2949019

[pbi12681-bib-0051] Szabados, L. and Savoure, A. (2010) Proline: a multifunctional amino acid. Trends Plant Sci. 15, 89–97. 20036181 10.1016/j.tplants.2009.11.009

[pbi12681-bib-0052] Tanaka, N. , Matsuoka, M. , Kitano, H. , Asano, T. , Kaku, H. and Komatsu, S. (2006) gid1, a gibberellin‐insensitive dwarf mutant, shows altered regulation of probenazole‐inducible protein (PBZ1) in response to cold stress and pathogen attack. Plant, Cell Environ. 29, 619–631. 17080612 10.1111/j.1365-3040.2005.01441.x

[pbi12681-bib-0053] Thimm, O. , Blasing, O. , Gibon, Y. , Nagel, A. , Meyer, S. , Kruger, P. , Selbig, J. *et al*. (2004) MAPMAN: a user‐driven tool to display genomics data sets onto diagrams of metabolic pathways and other biological processes. Plant J. 37, 914–939. 14996223 10.1111/j.1365-313x.2004.02016.x

[pbi12681-bib-0054] Wang, C. , Yang, Y. , Wang, H. , Ran, X. , Li, B. , Zhang, J. and Zhang, H. (2016) Ectopic expression of a cytochrome P450 monooxygenase gene PtCYP714A3 from *Populus trichocarpa* reduces shoot growth and improves tolerance to salt stress in transgenic rice. Plant Biotech. J. 14, 1838–1851. 10.1111/pbi.12544 PMC5069455 26970512

[pbi12681-bib-0055] Yamaguchi, S. (2008) Gibberellin metabolism and its regulation. Annu. Rev. Plant Biol. 59, 225–251. 18173378 10.1146/annurev.arplant.59.032607.092804

[pbi12681-bib-0056] Yang, X.C. and Hwa, C.M. (2008) Genetic modification of plant architecture and variety improvement in rice. Heredity 101, 396–404. 18716608 10.1038/hdy.2008.90

