# Supplementary material for: Ectopic expression of specific GA2 oxidase mutants promotes yield and stress tolerance in rice
Source: Plant Biotechnol J. 2017 Mar 23;15(7):850–64. doi: 10.1111/pbi.12681 (PMC5466439; doi:10.1111/pbi.12681)
Supplement: Supplementary file 1 — Figure S1. Phylogenetic tree analysis of GA2oxs in plants. Figure S2. Ectopic expression of GA2ox6 mutants alters plant height in rice. Figure S3. Plant heights are reduced, germination rates are unaltered, and shoot/root ratios are decreased in transgenic lines E140A, A141E and G343A. Figure S4. Treatment with exogenous GA3 restores normal height in transgenic lines A141E and G343A. Figure S5. Yields are increased in transgenic lines E140A and A141E in field trials. Figure S6. Grain weight and morphology and panicle weight and length in transgenic line A141E are similar to those in NT. Figure S7. Drought stress tolerance is enhanced in GA deficient transgenic rice. Figure S8. The size of bulliform cells is expanded in GA deficient transgenic rice. Figure S9. Plant volume, biomass and water content are increased in GA deficient transgenic rice. Figure S10. Reprogramming of GA‐regulated transcriptional networks in GA deficient transgenic rice. Figure S11. Expression of genes overrepresented in roots of GA deficient transgenic plants in response to abiotic and biotic stresses. Figure S12. Expression of genes overrepresented in shoots of GA deficient transgenic plants in response to abiotic stresses. Table S1. Gene names and accession numbers of GA2oxs from different plant species. Table S2. GA deficiency redirected several important transcriptional networks in roots. Table S3. GA deficiency redirected several important transcriptional networks in shoots. Table S4. Primers used for site‐directed mutagenesis, PCR and RT‐PCR analyses and plasmid constructions. [file PBI-15-850-s003.doc]

Supporting Information for

Ectopic expression of specific GA2 oxidase mutants promotes yield

and stress tolerance in rice

Shuen-Fang Lo^1,2^, Tuan-Hua David Ho^2,3,5^*, Yi-Lun Liu^1,2^, Mirng-Jier Jiang^1,2^,

Kun-Ting Hsieh^4^, Ku-Ting Chen^1^, Lin-Chih Yu^1^, Miig-Huey Lee^6^, Chi-yu Chen^6^, Tzu-Pi Huang^6^, Mikiko Kojima^7^, Hitoshi Sakakibara^7^,

Liang-Jwu Chen^2,4^*, and Su-May Yu^1,2,5^*

^1^ Institute of Molecular Biology, Academia Sinica, Nankang, Taipei 115, Taiwan, ROC.

^2^ Agricultural Biotechnology Center, National Chung Hsing University, Taichung 402, Taiwan, ROC.

^3^ Institute of Plant and Microbial Biology, Academia Sinica, Taipei 115, Taiwan, ROC.

1. Institute of Molecular Biology, National Chung Hsing University, Taichung 402, Taiwan, ROC.

^5^ Department of Life Sciences, National Chung Hsing University, Taichung 402, Taiwan, ROC

^6^. Department of Plant Pathology, National Chung Hsing University, Taichung 402, Taiwan, ROC

^7^ RIKEN Center for Sustainable Resource Science, Yokohama, Kanagawa 230-0045, Japan

Running title: Modified GA2ox promotes yield and stress tolerance

Key words: rice, gibberellin, GA 2 oxidase, plant architecture, yield, stress tolerance

* Co-corresponding authors:

Su-May Yu

Phone: 886-2-2788-2695

FAX: 886-2-2788-2695

e-mail : [sumay@imb.sinica.edu.tw](mailto:sumay@imb.sinica.edu.tw)

Liang-Jwu Chen

Phone: 886-4-2285-1885

FAX: 886-4-2287-4879

e-mail: [ljchen@nchu.edu.tw](mailto:ljchen@nchu.edu.tw)

Tuan-Hua David Ho

Phone: 886-2-2787-1090

FAX: 886-2-2782-1605

E-mail: [tho@gate.sinica.edu.tw](mailto:tho@gate.sinica.edu.tw); [ho@biology2.wustl.edu](mailto:ho@biology2.wustl.edu)


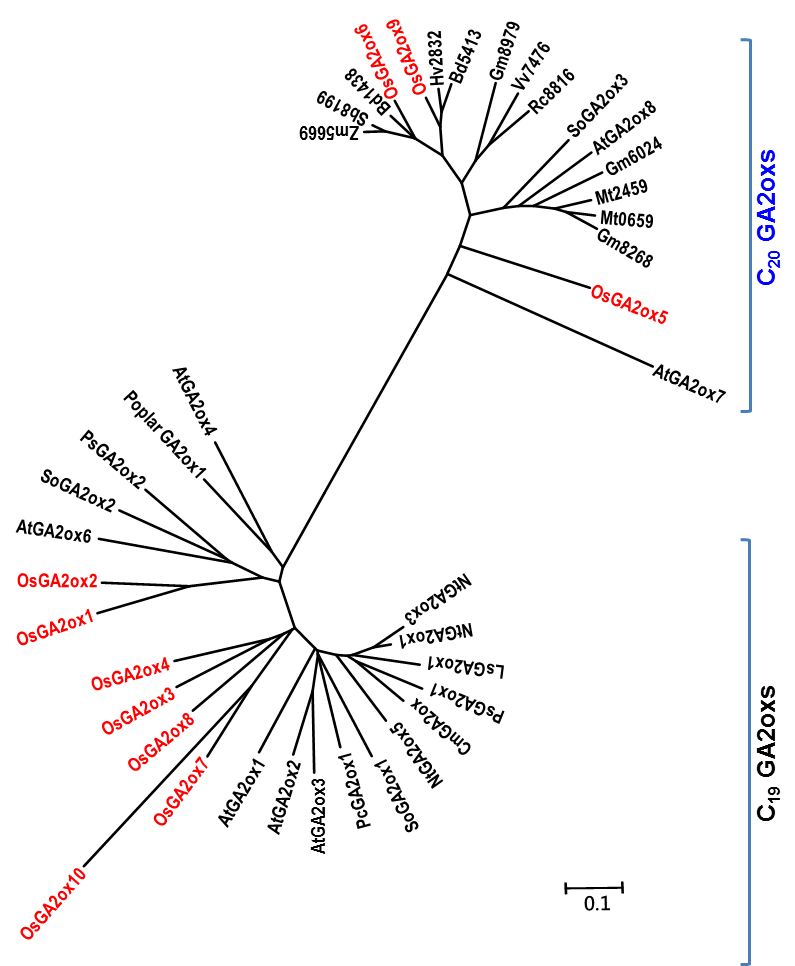


**Figure S1.** Phylogenetic tree analysis of GA2oxs in plants.

The evolutionary history was inferred using the Neighbor-Joining method . The optimal tree with the sum of branch length = 8.18143498 is shown. The tree is drawn to scale, with branch lengths in the same units as those of the evolutionary distances used to infer the phylogenetic tree. Evolutionary distances were computed using the Poisson correction method and are in the units of the number of amino acid substitutions per site. The analysis involved 41 amino acid sequences. All positions containing gaps and missing data were eliminated. There were a total of 214 positions in the final dataset. Evolutionary analyses were conducted in MEGA5 . Amino acid sequences of 41 GA2oxs were identified in eleven plant species. C_19_ and C_20_ GA2oxs were separated to two clades. The scale value of 0.1 indicates 0.1 amino acid substitution per site. Plant species: At, *Arabidopsis thaliana*; Bd, *Brachypodium distachyon*; Cm, *Cucurbita maxima*; Gm, *Glycine max*; Hv, *Hordeum vulgare*; Ls, *Lactuca sativa*; Mt, *Medicago truncatula*; Nt, *Nicotiana sylvestris*; Os, *Oryza sativa*; Pc, *Phaseolus coccineus*; PaPt, *Populus alba* x *P. tremuloides*; Ps, *Pisum sativum*; Rc, *Ricinus communis*; Sb, *Sorghum bicolor*; So, *Spinacia oleracea*; Vv, *Vitis vinifera*; Zm, *Zea mays*.


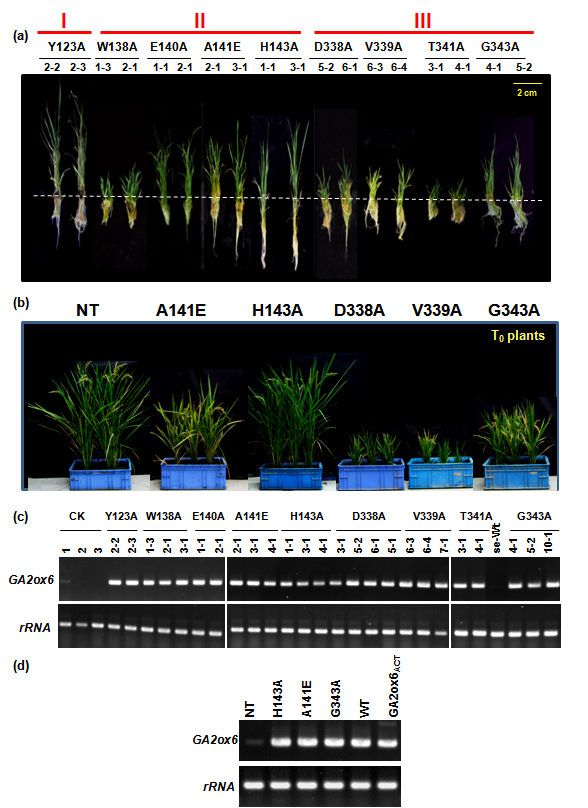


**Figure S2.** Ectopic expression of GA2ox6 mutants alters plant height in rice.

(a) Phenotypes of T0 transgenic rice seedlings overexpressing GA2ox6 mutants. The white dashed line indicates the position of shoot and root junction in seedlings. Motifs I, II and III denote the conserved 30 amino acids of C_20_ GA2oxs. (b) Phenotypes of mature T0 transgenic rice overexpressing GA2ox6 mutants. (c) GA2ox6 mutants are overexpressed at similar levels in independent T0 transgenic lines carrying various *Ubi:GA2ox6* mutant constructs. (d) GA2ox6 mutants are overexpressed at similar levels in T1 transgenic lines carrying various *Ubi:GA2ox6* mutant constructs. GA2ox6_ACT_ is GA2ox6 activated T-DNA mutant from the Taiwan Rice Insertional mutant (TRIM) population The accumulation of mRNA of GA2ox6 mutants in transgenic rice was determined by RT-PCR. The *18S rRNA* was used as an RNA loading control.


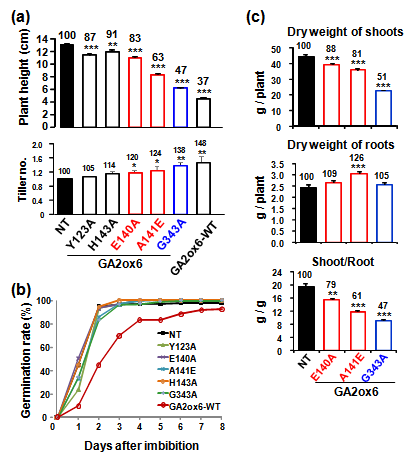


**Figure S3.** Plant heights are reduced, germination rates are unaltered, and shoot/root ratios are decreased in transgenic lines E140A, A141E and G343A.

T3 transgenic rice overexpressing various GA2ox6 mutant constructs. (a) Plant height and tiller number of 14-day-old plants. n= 15. (b) Rates of seed germination, n=154, 30, 30, 154, 54, 154, 154 for NT and transgenic lines Y123, E140, A141, H143, G343 and GA2ox6-WT, respectively. (c) One hundred and twenty-day-old plants were used in the experiment. The shoot/root ratio was calculated by dividing the shoot dry weight with the root dry weight. n=41, 77, 90, 93 for NT and transgenic lines E140, A141, and G343, respectively.


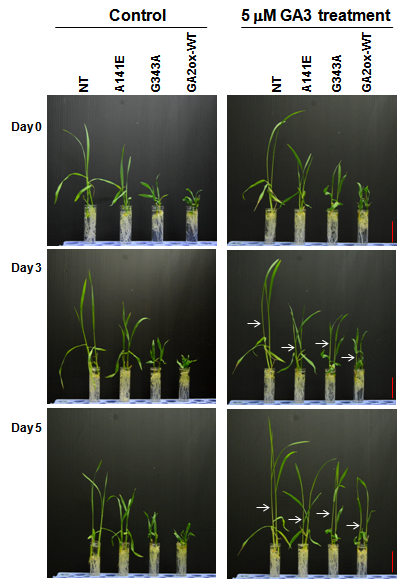


**Figure S4.**  Exogenous GA3 can restore the dwarfism morphology in transgenic lines A141E and G343A.

17-day-old seedlings were transferred to water w/ or w/o 5 μM GA3, the shoots of NT, A141E, G343A and GA2ox6-WT significantly elongated after 4 days of treatment; and the transgenic plants reached to similar plant height after 5 days of GA3 treatment; which demonstrate the various levels of dwarfism were caused by different extent of GA deficiency, and the dwarfism can be restored by exogenous GA supplement. Scare bar = 3 cm.


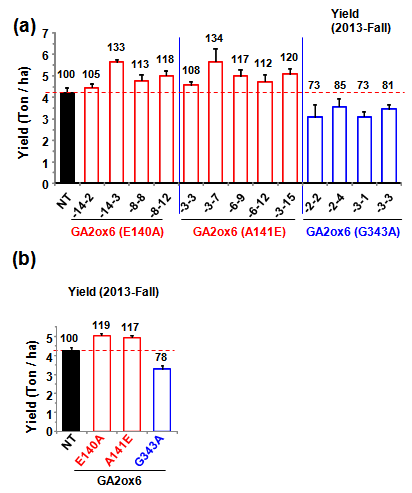


**Figure S5.** Yields are increased in transgenic lines E140A and A141E in field trials.

(a) Yield in various T3 homozygous transgenic lines in fall, 2013. (b) Average of yields in transgenic lines shown in (A). n = 32, 55, 54, and 30 for NT, A141E, G343A and WT GA2ox6, respectively. The red dashed line marks the yield in NT.

**
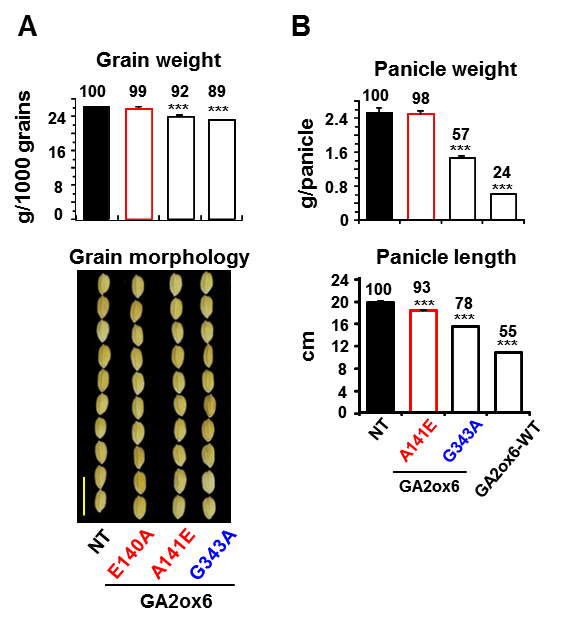
**

**Figure S6.** Grain weight and morphology and panicle weight and length in transgenic line A141E are similar to those in NT.

Thousand-grain weight, grain morphology, and panicle weight and length were measured or examined right after grain harvest.


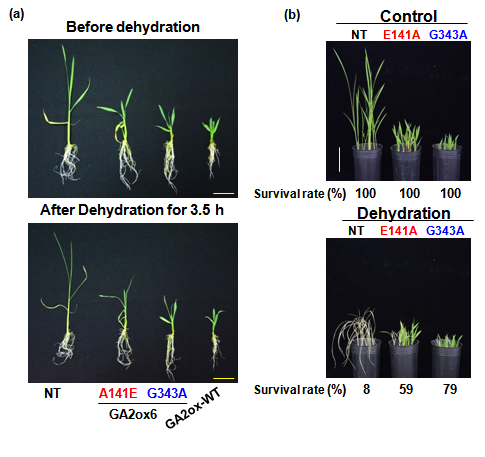


**Figure S7.** Drought stress tolerance is enhanced in GA deficient transgenic rice.

The following experiments were repeated twice with same results. (a) Twenty five-day-old plants before and after dehydration (air drying) for 3.5 h. Scale bar = 5 cm. (b) Six 4-day-old seedlings were transferred to soil in one pot, and cultivated with 1/3 Kimura solution for 20 days. Then plants were dehydrated for 11 days and re-watered for 3-days. Survival rates (%) were determined after recovery with water.

**
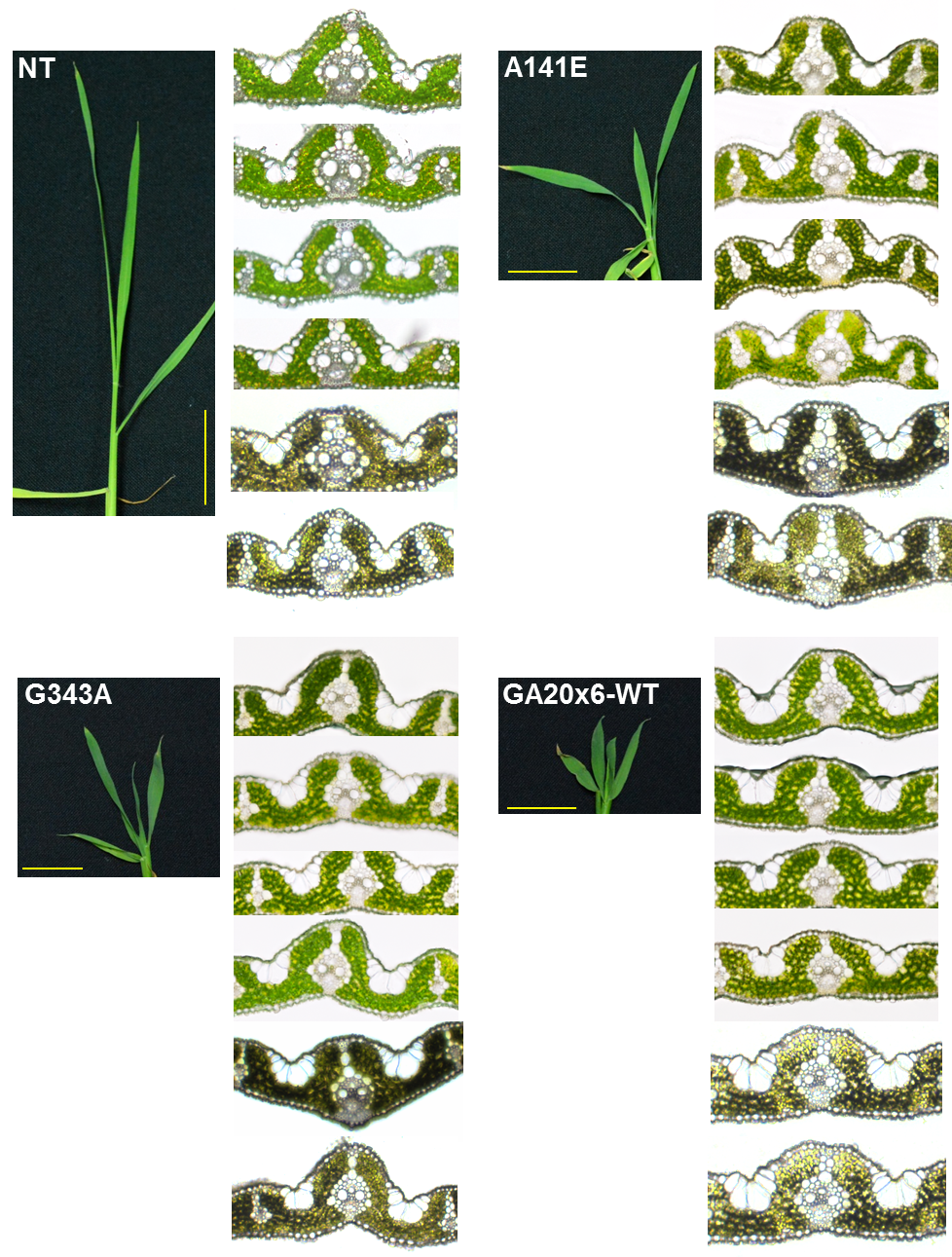
**

**Figure S8.** The size of bulliform cells is expanded in GA deficient transgenic rice.

Cross sections of leaves show bulliform cells flanking the large vein in rice. Bar scale: 100 μm.

**
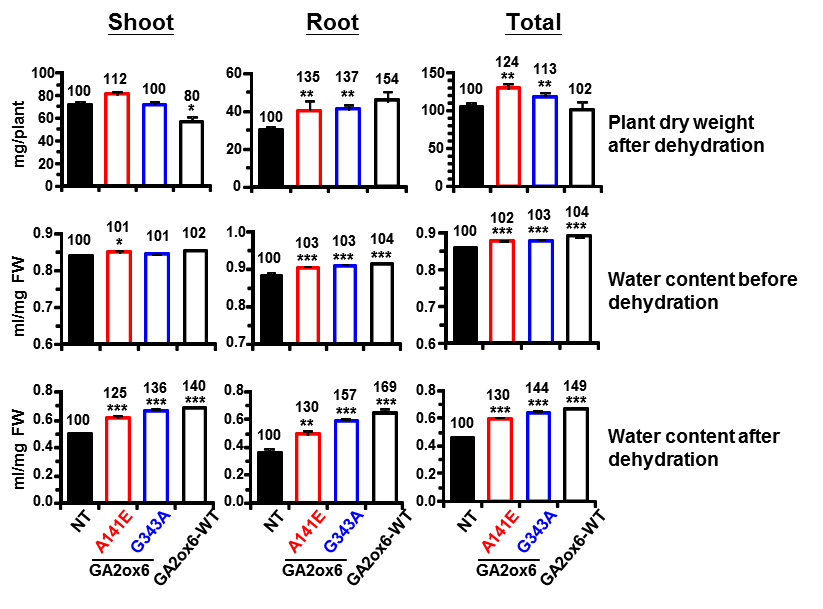
**

**Figure S9.**  Plant volume, biomass and water content are increased in GA deficient transgenic rice.

Seventeen-day-old seedlings were used for determination of plant dried weight and water contents before and after dehydration (air drying) for 3.5 h. n=10, 11, 11, 10 for NT and lines A141E, G343A, and GA2ox6-WT, respectively.


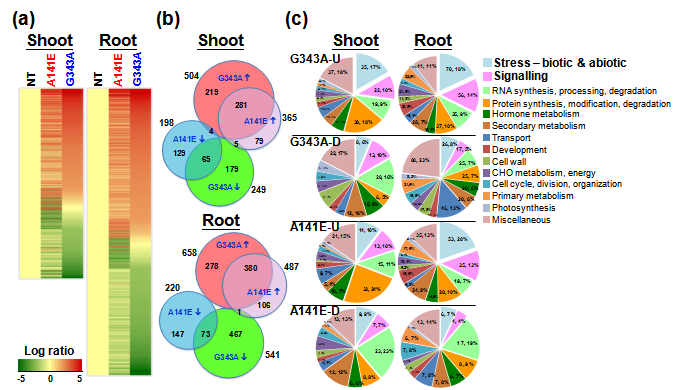


**Figure S10.** Reprograming of GA-regulated transcriptional networks in GA deficient transgenic rice.

(a) Heat map and (b) Venn diagram were constructed based on significantly different transcript abundance, with signal ratio change > 3 fold in the microarray analysis, in shoots and roots in A141E and G343A transgenic rice. Numbers outside the Venn diagram indicate total number of genes up- or down-regulated in each of the two transgenic lines. (c) The pie diagram shows the relative abundance of enriched transcripts that fall into different functional categories. U, up-regulated; D, down-regulated.

**
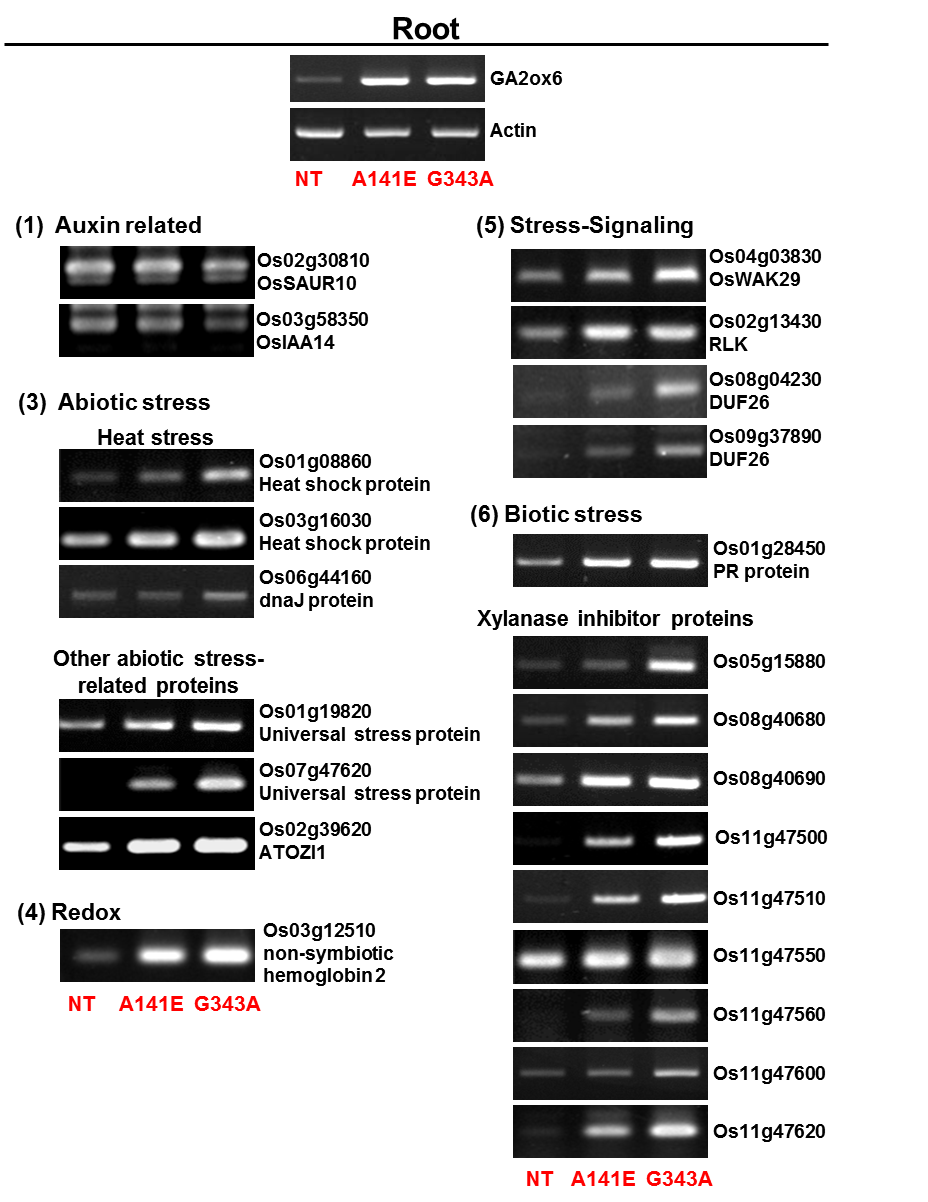
**

**Figure S11.** Expression of genes overrepresented in roots of GA deficient transgenic plants in response to abiotic and biotic stresses.

Total RNA was extracted from roots of 17-day-old seedlings and mRNA accumulation was determined by RT-PCR analysis. All genes are up-regulated except group 1 auxin-related genes which are down-regulated.

**
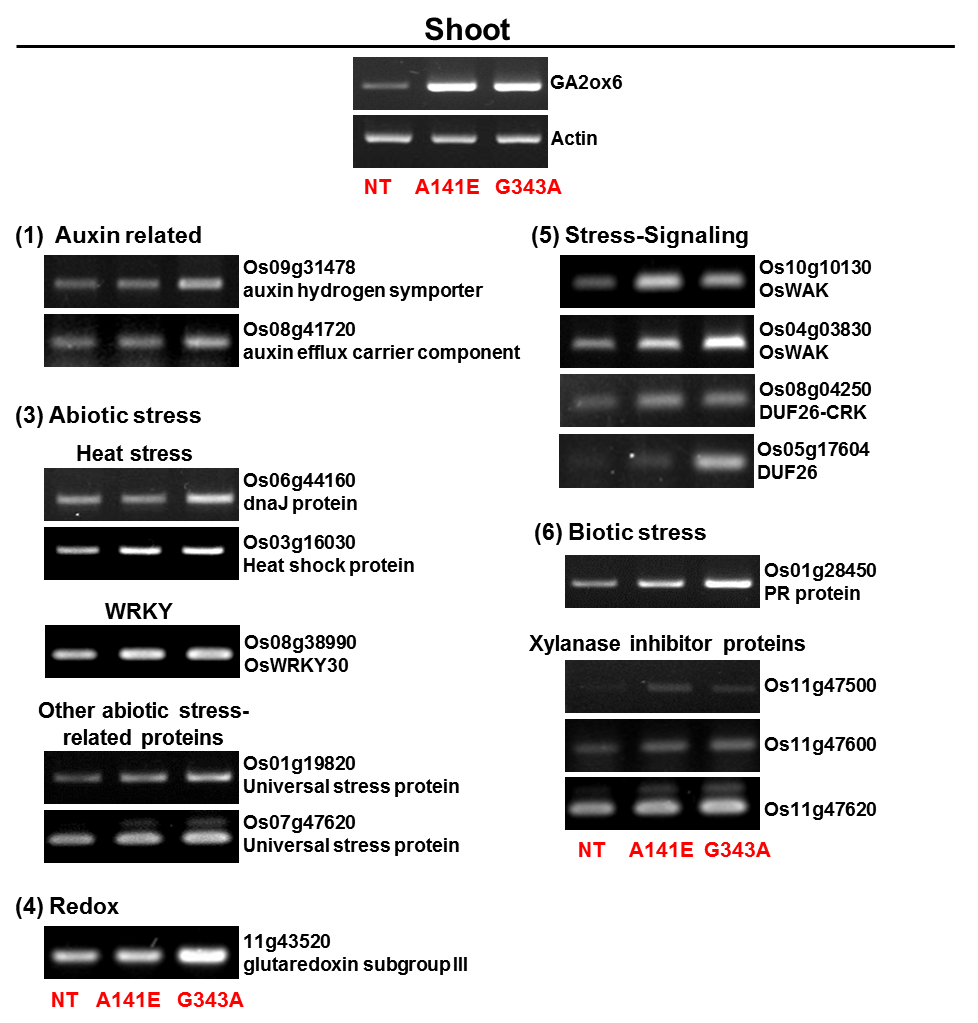
**

**Figure S12.** Expression of genes overrepresented in shoots of GA deficient transgenic plants in response to abiotic stresses.

Total RNA was extracted from shoots of 17-day-old seedlings and mRNA accumulation was determined by RT-PCR analysis.

**Table S1.** Gene names and accession numbers of GA2oxs from different plant species.

| Gene name | Type of GA2oxs | Species | Accession Number |
| --- | --- | --- | --- |
| *AtGA2ox1* | **C_19_** | *Arabidopsis thaliana* | AJ132435 |
| *AtGA2ox2* | **C_19_** | *Arabidopsis thaliana* | AJ132436 |
| *AtGA2ox3* | **C_19_** | *Arabidopsis thaliana* | AJ132437 |
| *AtGA2ox4* | **C_19_** | *Arabidopsis thaliana* | AY859740 |
| *AtGA2ox6* | **C_19_** | *Arabidopsis thaliana* | AY859741 |
| *AtGA2ox7* | **C_20_** | *Arabidopsis thaliana* | NM103976 |
| *AtGA2ox8* | **C_20_** | *Arabidopsis thaliana* | NM118239 |
| *Bd-1438* | **C_20_** | *Brachypodium distachyon* | XP_003581438 |
| *Bd-5413* | **C_20_** | *Brachypodium distachyon* | XP_003575413 |
| *CmGA2ox* | **C_19_** | *Cucurbita maxima* | AJ302041 |
| *Gm-6024* | **C_20_** | *Glycine max* | XP_003556024 |
| *Gm-8268* | **C_20_** | *Glycine max* | XP_003538628 |
| *Gm-8979* | **C_20_** | *Glycine max* | XP_003538979 |
| *Hv-2832* | **C_20_** | *Hordeum vulgare* | BAJ92832 |
| *LsGA2ox1* | **C_19_** | *Lactuca sativa* | AB031206 |
| *Mt-0659* | **C_20_** | *Medicago truncatula* | XP_003610659 |
| *Mt-2459* | **C_20_** | *Medicago truncatula* | ABD32459 |
| *NtGA2ox1* | **C_19_** | *Nicotiana sylvestris* | AB125232 |
| *NtGA2ox3* | **C_19_** | *Nicotiana sylvestris* | EF471117 |
| *NtGA2ox5* | **C_19_** | *Nicotiana sylvestris* | EF471118 |
| *OsGA2ox1* | **C_19_** | *Oryza sativa* | EAY96632 |
| *OsGA2ox2* | **C_19_** | *Oryza sativa* | BAC16751 |
| *OsGA2ox3* | **C_19_** | *Oryza sativa* | NP_001044292 |
| *OsGA2ox4* | **C_19_** | *Oryza sativa* | EEE64351 |
| *OsGA2ox5* | **C_20_** | *Oryza sativa* | NP_001058690 |
| *OsGA2ox6* | **C_20_** | *Oryza sativa* | NP_001053341 |
| *OsGA2ox7* | **C_19_** | *Oryza sativa* | NP_001042364 |
| *OsGA2ox8* | **C_19_** | *Oryza sativa* | NP_001056311 |
| *OsGA2ox9* | **C_20_** | *Oryza sativa* | EEE57418 |
| *OsGA2ox10* | **C_19_** | *Oryza sativa* | AAT01379 |
| *PcGA2ox1* | **C_19_** | *Phaseolus coccineus* | AJ132438 |
| *Poplar GA2ox1* | **C_19_** | *Populus alba x P. tremuloides* | AY392094 |
| *PsGA2ox1* | **C_19_** | *Pisum sativum* | AF056935 |
| *PsGA2ox2* | **C_19_** | *Pisum sativum* | AF100954 |
| *Rc-8816* | **C_20_** | *Ricinus communis* | XP_002518816 |
| *Sb-8199* | **C_20_** | *Sorghum bicolor* | XP_002448199 |
| *SoGA2ox1* | **C_19_** | *Spinacia oleracea* | AF506281 |
| *SoGA2ox2* | **C_19_** | *Spinacia oleracea* | AF506282 |
| *SoGA2ox3* | **C_20_** | *Spinacia oleracea* | AY935713 |
| *Vv-7476* | **C_20_** | *Vitis vinifera* | CBI27476 |
| *Zm-5669* | **C_20_** | *Zea mays* | ACN35669 |

**Table S2.** GA deficiency redirected several important transcriptional networks in roots.


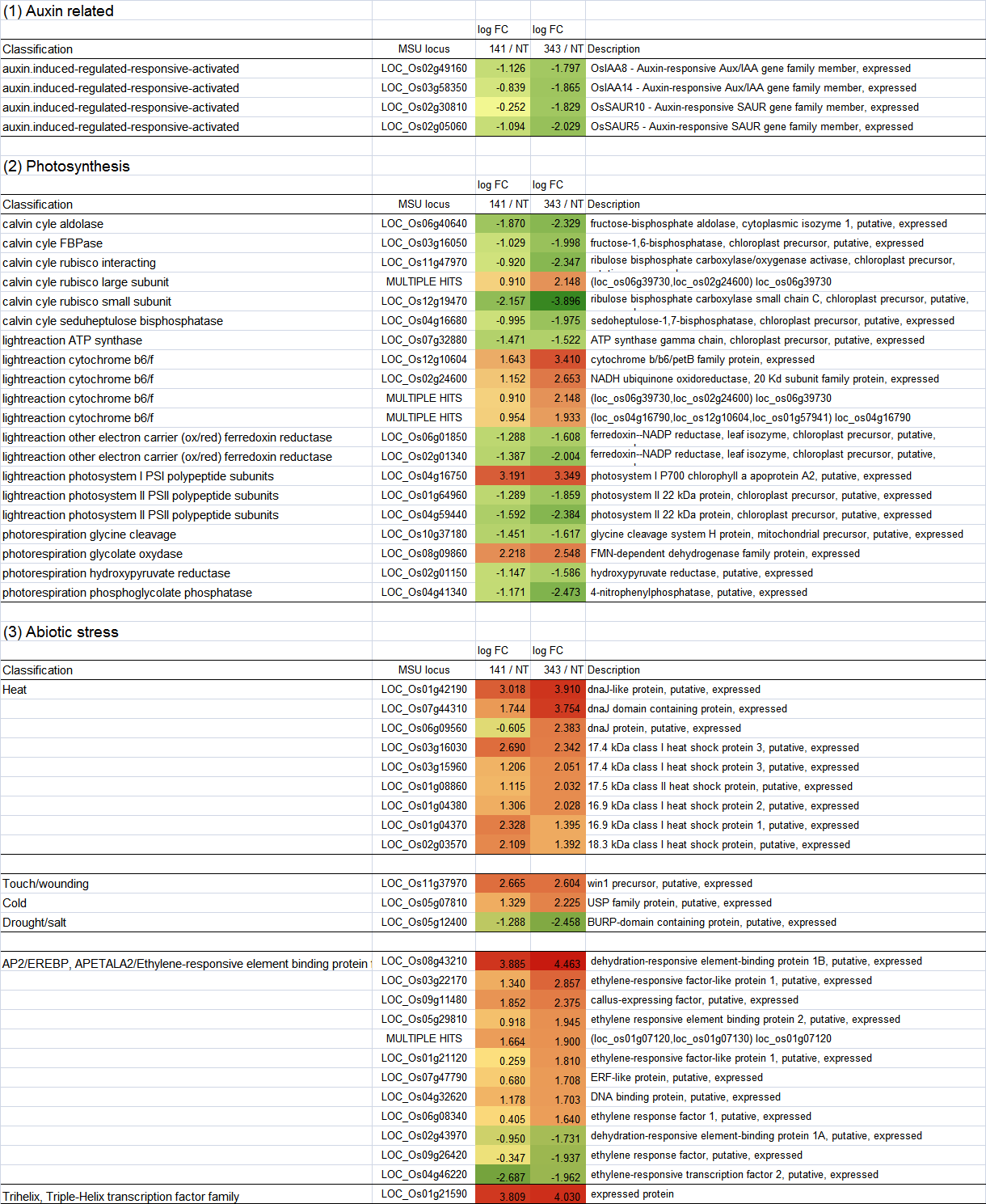


**To be continued**


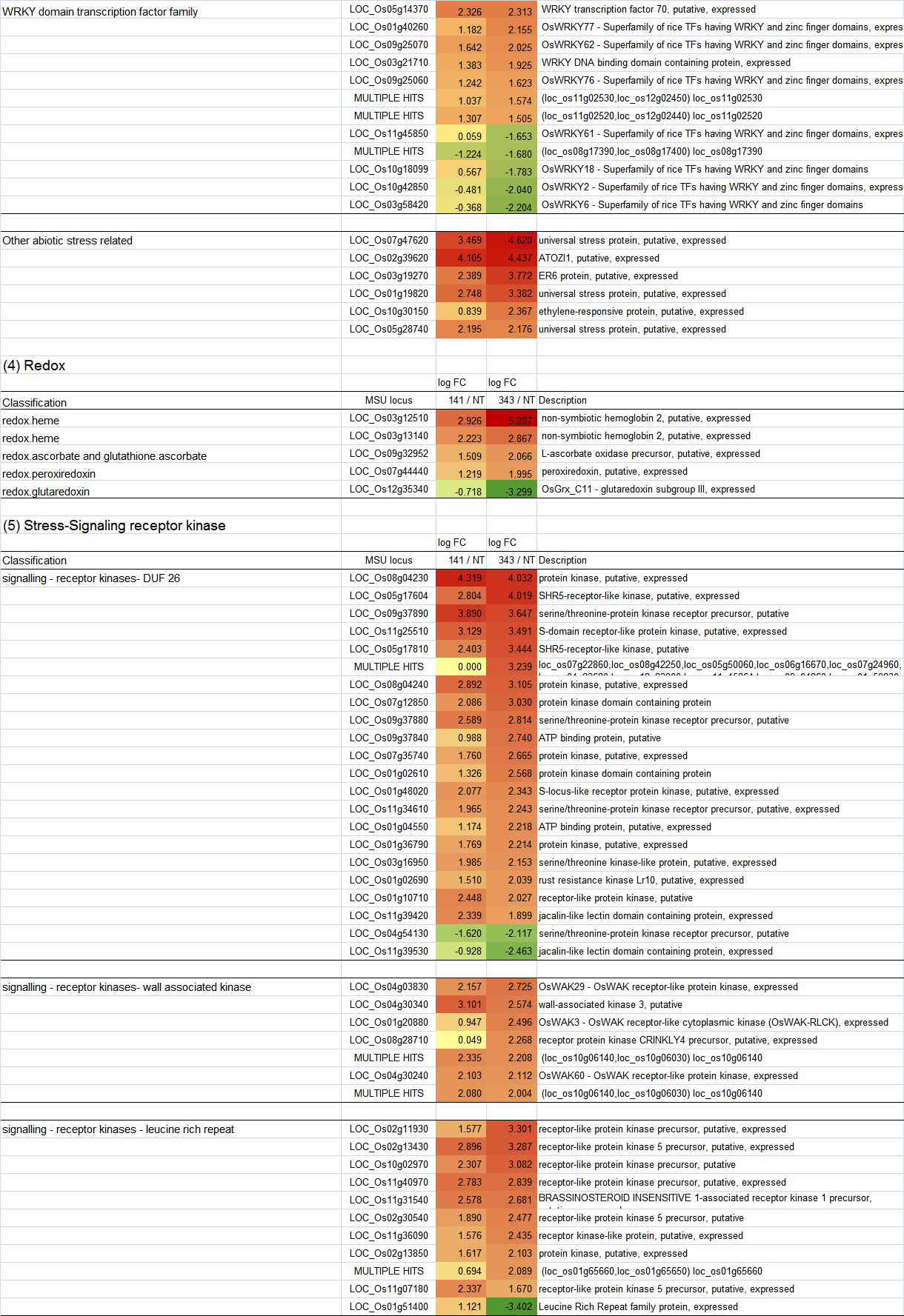


**To be continued**


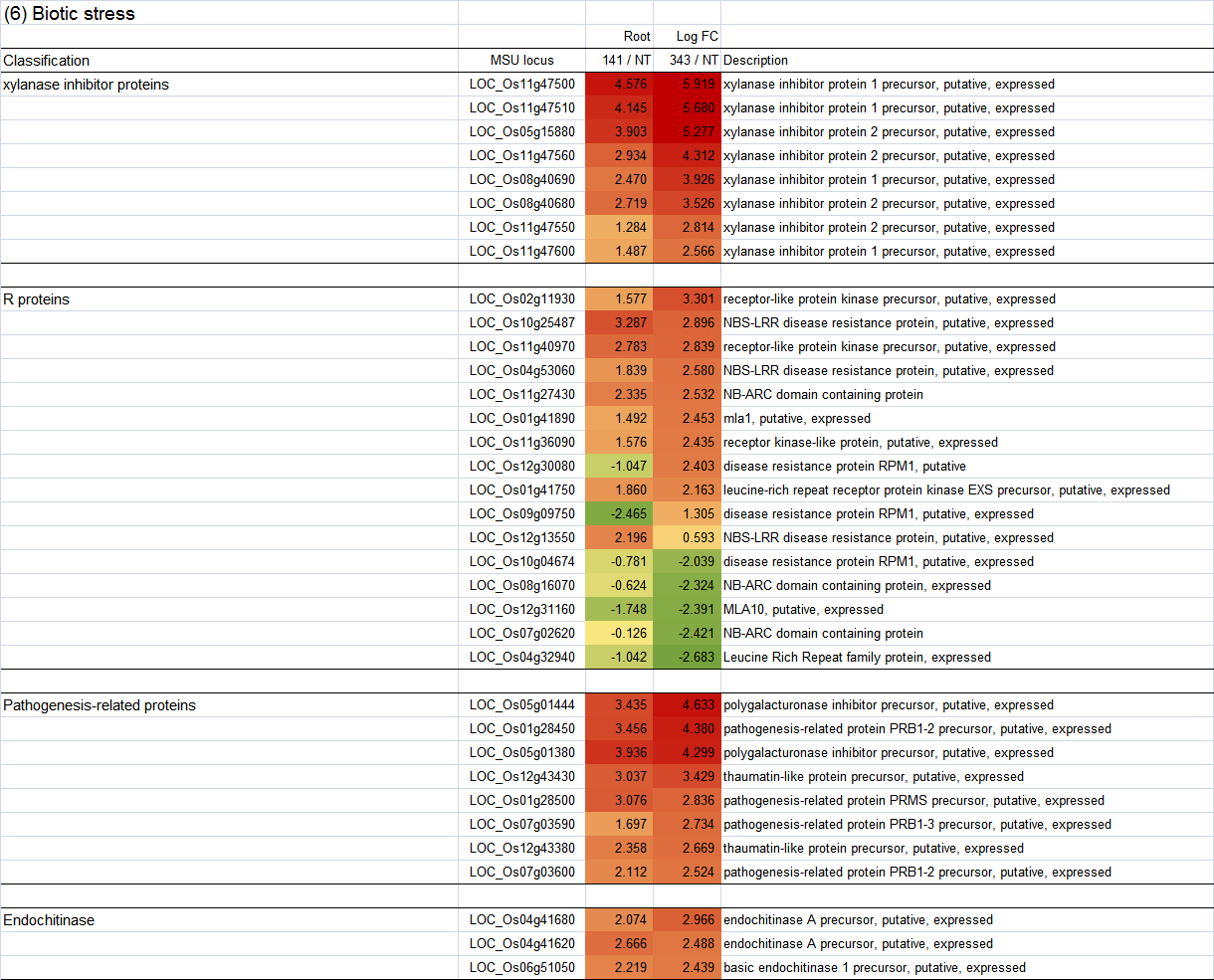


**Table S3.** GA deficiency redirected several important transcriptional networks in shoots.


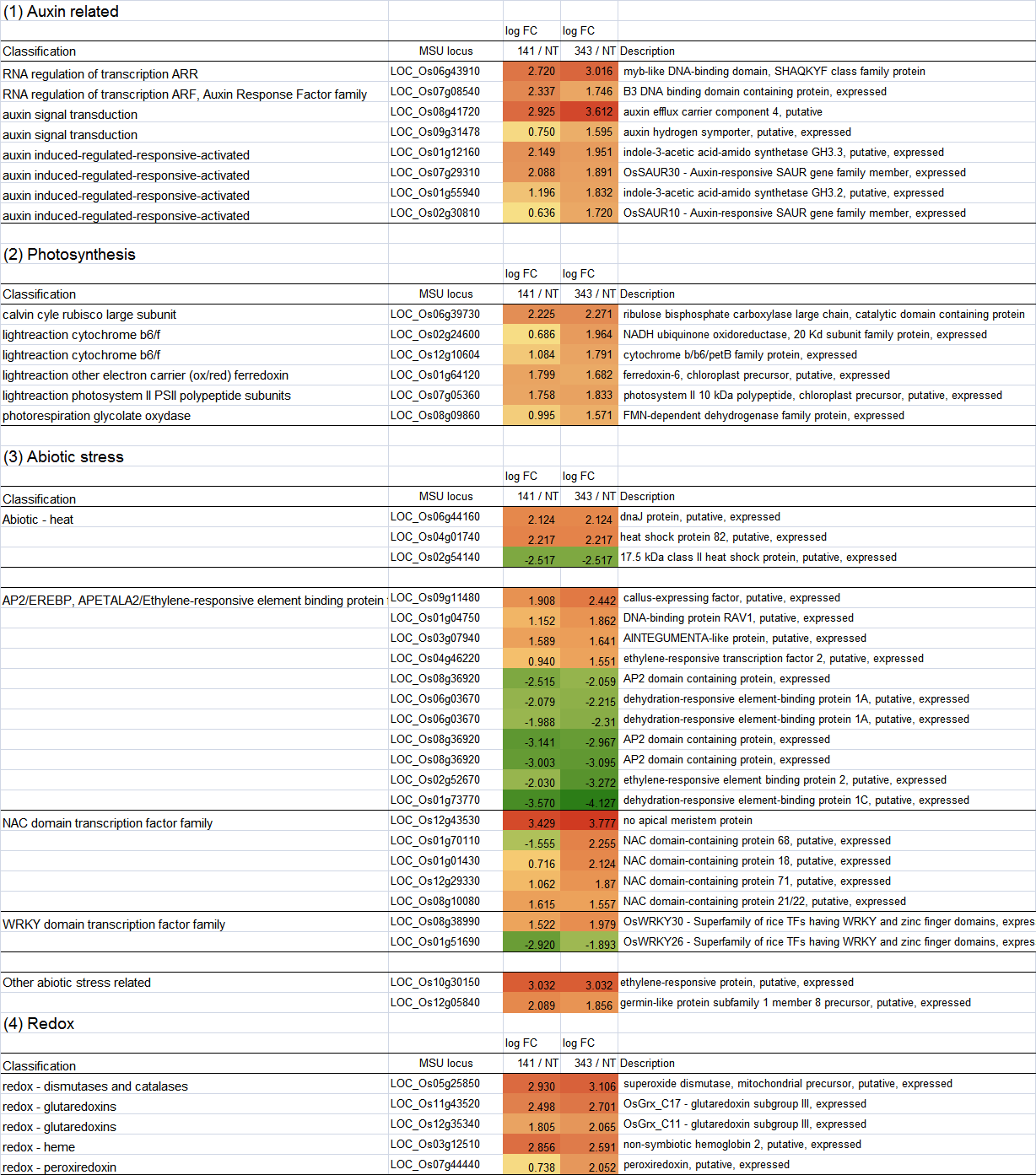


**To be continued**


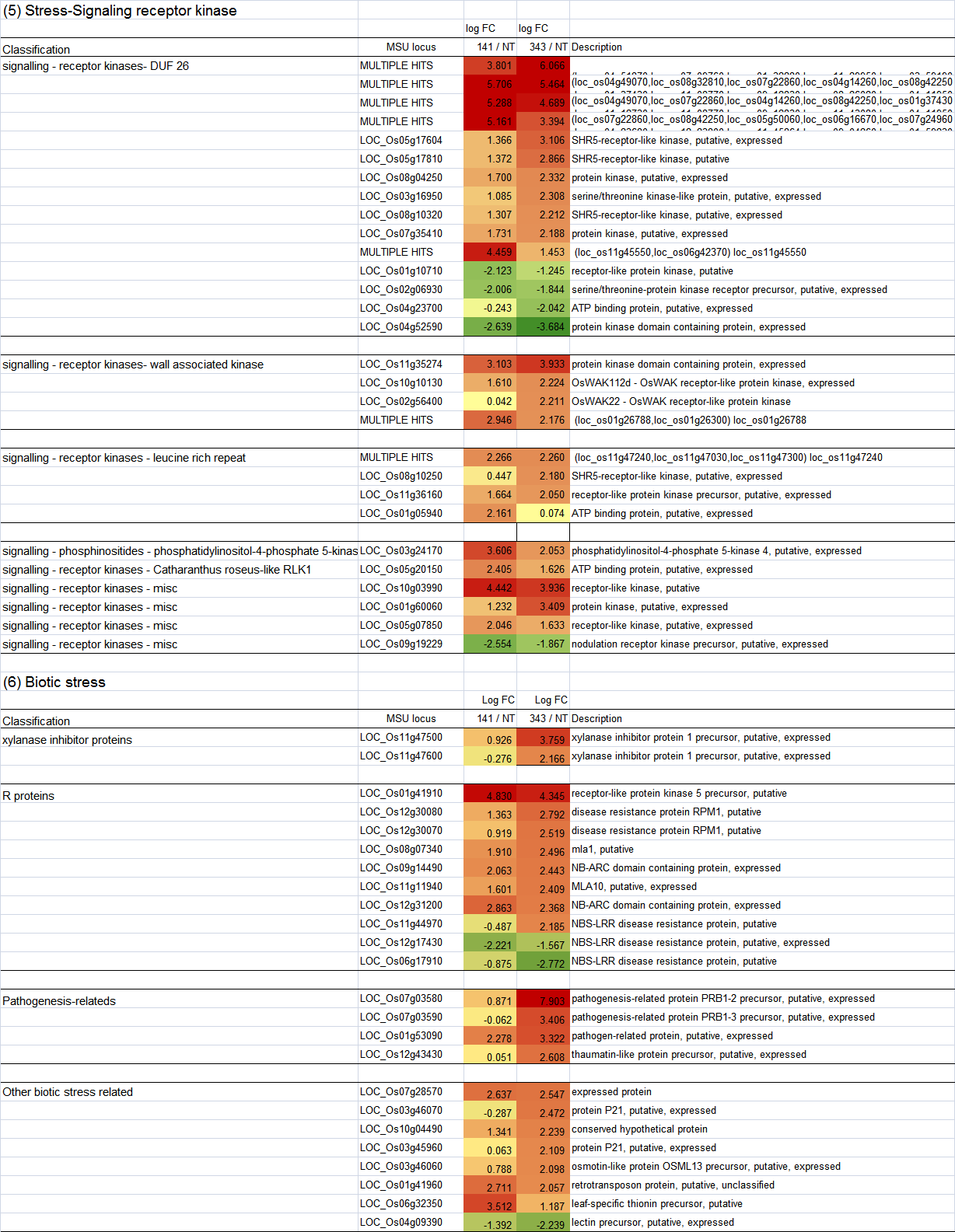


**Table S4.** Primers used for **site-directed mutagenesis**, PCR and RT-PCR analyses and plasmid constructions.

| Primers | Sequence | Gene |
| --- | --- | --- |
| **Primers for site-directed mutagenesis** | | |
| Y123A-F | 5’- CTCAACGGCTCG**GCT**CGGTGGGGCAAC -3’ | |
| Y123A-R | 5’- GTTGCCCCACCG**AGC**CGAGCCGTTGAG -3’ | |
| W138A-F | 5’- CCACCTCTCG**GCT**TCGGAGGCGTTC -3’ | |
| W138A-R | 5’- GAACGCCTCCGA**AGC**CGAGAGGTGG -3’ | |
| E140A-F | 5’- CTCTCGTGGTCG**GCT**GCGTTCCACGTC-3’ | |
| E140A-R | 5’- GACGTGGAACGC**AGC**CGACCACGAGAG-3’ | |
| A141E-F | 5’- CGTGGTCGGAG**GAG**TTCCACGTCCC-3’ | |
| A141E-R | 5’- GGGACGTGGAA**CTC**CTCCGACCACG-3’ | |
| H143A-F | 5’- GTCGGAGGCGTTC**GCC**GTCCCGCTCG-3’ | |
| H143A-R | 5’- CGAGCGGGAC**GGC**GAACGCCTCCGAC -3’ | |
| D338A-F | 5’- GAAGGTGCAGGAA**GCC**GTCAGGACAAC-3’ | |
| D338A-R | 5’- GTTGTCCTGAC**GGC**TTCCTGCACCTTC-3’ | |
| V339A-F | 5’- GTGCAGGAAGAC**GCC**AGGACAACCG -3’ | |
| V339A-R | 5’- CGGTTGTCCT**GGC**GTCTTCCTGCAC-3’ | |
| T341A-F | 5’- GAAGACGTCA**GGG**CAACCGGGAAAAAG-3’ | |
| T341A-R | 5’- CTTTTTCCCGGTTG**CCC**TGACGTCTTC-3’ | |
| G343A-F | 5’- CAGGACAACC**GCC**AAAAAGATTGGCCTC-3’ | |
| G343A-R | 5’- GAGGCCAATCTTTTT**GGC**GGTTGTCCTG-3’ | |
|  |  | |
| **Primers for RT-PCR- analysis of gene expression** | | |
| GA2ox6-F | 5’- GACGACGTGCTTCCTGCGGCTCAA-3’ | *OsGA2ox6* (389 bp) |
| GA2ox6-R | 5’- CTTCCTGCACCTTCTTCCTGTA-3’ |  |
| 18S-F | 5’- CCTCGTGCCCCTATCAACTT-3’ | *18S rRNA* (201 bp) |
| 18S-R | 5’- GACACTAAAGCGCCCGGTAT-3’ |  |
| 01g08860-F | 5’-GAGAGCGCCATGTTCGG-3’ | 259 bp |
| 01g08860-R | 5’-GCAGCCTCTCCTCCTCCA-3’ |  |
| 01g19820-F | 5’-TGGCGTCGTCTGTTCCCG-3’ | 269 bp |
| 01g19820-R | 5’-CATCACGTCCGAGGTCATC-3’ |  |
| 01g28450-F | 5’-TACGACTACGCCTCCAACAGC-3’ | 296 bp |
| 01g28450-R | 5’- GACCGTGAAGGCGTGGAT3’ |  |
| 02g13430-F | 5’-AGCGGTACAACAATGGTGGC-3’ | 192 bp |
| 02g13430-R | 5’-CGCTATGTGTTCCGCTATGA-3’ |  |
| 02g39620-F | 5’-TGGCGACCAAGTACATCATC-3’ | 222 bp |
| 02g39620-R | 5’-TGACGATGAAGTTCTGGCG-3’ |  |
| 03g16030-F | 5’-GGCAGCATCTTCCCGTCCT-3’ | 304 bp |
| 03g16030-R | 5’-TGGACGCCTTGATCTGCT-3’ |  |
| 04g03830-F | 5’-GACCTGTGCTGCTGTGCCT-3’ | 244 bp |
| 04g03830-R | 5’-GCGGATAGAAGAGCGAATGG-3’ |  |
| 05g15880-F | 5’-CAACTTCGGAGGCGTCAT-3’ | 236 bp |
| 05g15880-R | 5’-GATTACATACATTACCCAACATAC-3’ |  |
| 06g44160-F | 5’-GAGGGATGCTGTGCGTGTC-3’ | 325 bp |
| 06g44160-R | 5’-TACTCCAGCCTCTCCTCGG-3’ |  |
| 07g47620-F | 5’-GGAGCAGGGGAAGACGG-3’ | 203 bp |
| 07g47620-R | 5’-CGTATCTCACCACCTCTCCAG-3’ |  |
| 08g04230-F | 5’-AGGCAGTAGGAAAGGCGACG-3’ | 223 bp |
| 08g04230-R | 5’-TAGTCAATCTGGCACCCCTG-3’ |  |
| 08g04250-F | 5’-TCGTCTCATTGGTTGCCTTG-3’ | 228 bp |
| 08g04250-R | 5’-CGTAGAAGACCTCGGAGCC-3’ |  |
| 08g38990-F | 5’-GCACCACAGGCTACCAATG-3’ | 244 bp |
| 08g38990-R | 5’-CACCTCTCGGCAGCACAAG-3’ |  |
| 08g40680-F | 5’-TGTATATAGGGTTGGTGGCG-3’ | 249 bp |
| 08g40680-R | 5’-TCCATCACAACATTACAAACACG-3’ |  |
| 08g40690-F | 5’-CGCAGTGGAACAGGTGGTC-3’ | 305 bp |
| 08g40690-R | 5’-CACGCACGGATTGGACTGA-3’ |  |
| 08g41720-F | 5’-GGCAGCAGGAGAGGGTGAT-3’ | 229 bp |
| 08g41720-R | 5’-ATAACCGCCGTGCTGAGTA-3’ |  |
| 09g31040-F | 5’-GACGGCAATGGCACGGT-3’ | 285 bp |
| 09g31040-R | 5’-GGTTTCTGCCGTGTGCCAA-3’ |  |
| 09g31478-F | 5’-CTATTGCTTCAGTACGCCGTGC-3’ | 391 bp |
| 09g31478-R | 5’-ACACGAACGCCATGAATTGC-3’ |  |
| 09g37890-F | 5’-TGATAGGTGGTAAAGAAGTTGCTG-3’ | 370 bp |
| 09g37890-R | 5’-GCTATCTTGGGTCTCATCTCTGC-3’ |  |
| 10g10130-F | 5’-GCTTACCACGGGACATTTCAA-3’ | 232 bp |
| 10g10130-R | 5’-GGACACCAAACCATCACACC-3’ |  |
| 11g31550-F | 5’-GCGAATAATAATCTGGATGGCA-3’ | 317 bp |
| 11g31550-R | 5’-CAGAGGACTAGAAACAGGGGAAC-3’ |  |
| 11g31560-F | 5’-CACCTTGGATGAACTGAACG-3’ | 244 bp |
| 11g31560-R | 5’-CAGGCATATGCTCCACAGGG-3’ |  |
| 11g37970-F | 5’-ATGGTGGTGGCGCTCCTC-3’ | 250 bp |
| 11g37970-R | 5’-TCTTCACCTGGATACACTTGCC-3’ |  |
| 11g43520-F | 5’-AACCGGGTCATGTCGCTCCA-3’ | 198 bp |
| 11g43520-R | 5’-CAACTTCACCATTACACCACTCG-3’ |  |
| 11g47500-F | 5’-ATCATCTGCCACCGCCGT-3’ | 258 bp |
| 11g47500-R | 5’-TGGCAGTGGCGGATGTC-3’ |  |
| 11g47510-F | 5’-CGCAGTGGAACAGGTGGTC-3’ | 318 bp |
| 11g47510-R | 5’-CATGACACCGTGATTACTGCGA-3’ |  |
| 11g47560-F | 5’-ACCTGTCGCCCAAGCCACT-3’ | 266 bp |
| 11g47560-R | 5’-CCTTATTTAATTGCGACATGGG-3’ |  |
| 11g47560-F | 5’-TTCCTGACGGTGGTGGC | 335 bp |
| 11g47560-R | 5-‘ATAAAACTTTGCACCGTCTCG-3’ |  |
| 11g47600-F | 5’-ACTGGGAGGACTGGGACG-3’ | 276 bp |
| 11g47600-R | 5’-TCCGATGAAGCAGCAATGG-3’ |  |
| 11g47620-F | 5’-GGAGCAGGGGAAGACGG-3’ | 203 bp |
| 11g47620-R | 5’-CGTATCTCACCACCTCTCCAG-3’ |  |

**Supporting Video Legends**

**Video S1:** Transgenic rice over-expressing WT and mutant GA2ox6 recovered faster from dehydration than NT.

Twenty five-day-old plants were air-dried for 6 h and recovered in water for 9 h. Leaves of all plants rolled during dehydration (air drying) in empty tubes. Leaves opened rapidly in transgenic rice but much slowly in NT after plants were rehydrated with water filled in tubes.

**Video S2:** Transgenic rice over-expressing WT and mutant GA2ox6 recovered better from osmotic stress than NT.

Twenty five-day-old plants were treated with 30% of PEG6000 for 4.5 h and recovered in water for 6 h. Two top young leaves of NT and lines A141E and G343A wilted, but of line GA2ox6-WT did not show apparent wilting, during the treatment. Same two top leaves of lines A141E and G343A expanded and continued to grow, while the top youngest leaf of NT never expanded and die, after plants were recovered in water.

**References**

Saitou, N. and Nei, M. (1987) The neighbor-joining method: a new method for reconstructing phylogenetic trees. *Mol Biol Evol* **4**, 406-425.

Tamura, K., Peterson, D., Peterson, N., Stecher, G., Nei, M. and Kumar, S. (2011) MEGA5: molecular evolutionary genetics analysis using maximum likelihood, evolutionary distance, and maximum parsimony methods. *Mol Biol Evol* **28**, 2731-2739.

Zuckerkandl, E. and Pauling, L. (1965) Molecules as documents of evolutionary history. *J. Theor. Biol.* **8**, 357-366.
